# Multi-scale computational study of the Warburg effect, reverse Warburg effect and glutamine addiction in solid tumors

**DOI:** 10.1371/journal.pcbi.1006584

**Published:** 2018-12-07

**Authors:** Mengrou Shan, David Dai, Arunodai Vudem, Jeffrey D. Varner, Abraham D. Stroock

**Affiliations:** 1 Robert Frederick Smith School of Chemical and Biomolecular Engineering, Cornell University, Ithaca, New York, United States of America; 2 Kavli Institute at Cornell for Nanoscale Science, Cornell University, Ithaca, New York, United States of America; Ecole Polytechnique Fédérale de Lausanne, SWITZERLAND

## Abstract

Cancer metabolism has received renewed interest as a potential target for cancer therapy. In this study, we use a multi-scale modeling approach to interrogate the implications of three metabolic scenarios of potential clinical relevance: the Warburg effect, the reverse Warburg effect and glutamine addiction. At the intracellular level, we construct a network of central metabolism and perform flux balance analysis (FBA) to estimate metabolic fluxes; at the cellular level, we exploit this metabolic network to calculate parameters for a coarse-grained description of cellular growth kinetics; and at the multicellular level, we incorporate these kinetic schemes into the cellular automata of an agent-based model (ABM), iDynoMiCS. This ABM evaluates the reaction-diffusion of the metabolites, cellular division and motion over a simulation domain. Our multi-scale simulations suggest that the Warburg effect provides a growth advantage to the tumor cells under resource limitation. However, we identify a non-monotonic dependence of growth rate on the strength of glycolytic pathway. On the other hand, the reverse Warburg scenario provides an initial growth advantage in tumors that originate deeper in the tissue. The metabolic profile of stromal cells considered in this scenario allows more oxygen to reach the tumor cells in the deeper tissue and thus promotes tumor growth at earlier stages. Lastly, we suggest that glutamine addiction does not confer a selective advantage to tumor growth with glutamine acting as a carbon source in the tricarboxylic acid (TCA) cycle, any advantage of glutamine uptake must come through other pathways not included in our model (e.g., as a nitrogen donor). Our analysis illustrates the importance of accounting explicitly for spatial and temporal evolution of tumor microenvironment in the interpretation of metabolic scenarios and hence provides a basis for further studies, including evaluation of specific therapeutic strategies that target metabolism.

## Author summary

Cancer metabolism is an emerging hallmark of cancer. In the past decade, a renewed focus on cancer metabolism has led to several distinct hypotheses describing the role of metabolism in cancer. To complement experimental efforts in this field, a scale-bridging computational framework is needed to allow rapid evaluation of emerging hypotheses in cancer metabolism. In this study, we present a multi-scale modeling platform and demonstrate the distinct outcomes in population-scale growth dynamics under different metabolic scenarios: the Warburg effect, the reverse Warburg effect and glutamine addiction. Within this modeling framework, we confirmed population-scale growth advantage enabled by the Warburg effect, provided insights into the symbiosis between stromal cells and tumor cells in the reverse Warburg effect and argued that the anaplerotic role of glutamine is not exploited by tumor cells to gain growth advantage under resource limitations. We point to the opportunity for this framework to help understand tissue-scale response to therapeutic strategies that target cancer metabolism while accounting for the tumor complexity at multiple scales.

## Introduction

Cancer remains one of the leading causes of death worldwide. A central challenge in understanding and treating cancer comes from its multi-scale nature, with interacting defects at the molecular, cellular and tissue scales. Specifically, the molecular profile at the intracellular level, behavior at the single-cell level and the interactions between tumor cells and the surrounding tissues all influence tumor progression and complicate extrapolation from molecular and cellular properties to tumor behavior [1–3]. Understanding the multi-scale responses of cancer to microenvironmental stress could provide important new insights into tumor progression and aid the development of new therapeutic strategies [2]. Therefore, cancer must be studied and treated as a cellular ecology made up of individual cells and their microenvironment. This ecological view should account for the competition and cooperation of different molecular and cellular players, and for both the physical and biological characteristics of the environment in which tumor evolves. Such perspectives complement studies of the genetic drivers of tumor and potentially provide new bases for treating this disease [4].

Central to an ecological perspective of tumors is metabolism, the biochemical process by which cells derive energy and biomass from the nutrients available in their environment while excreting products of metabolism back to the environment. This exchange of metabolites impacts the distribution of resource in the environment and sets constraints on the availability of resources to individual cells [5]. Therefore, metabolism couples the behavior of individual cells to the characteristics–spatial-temporal organization and phenotypic make-up–of the full population. Recently, cancer metabolism has drawn renewed attention in the field of cancer biology [4,6]. Following the early observations of the unique tissue-scale metabolic profile of tumors made by Otto Warburg in the 1920s, discoveries of oncogenes and molecular cues in tumor-associated metabolic alterations have renewed the hope for therapeutic routes that target cancer metabolism [7].

**Fig 1:**
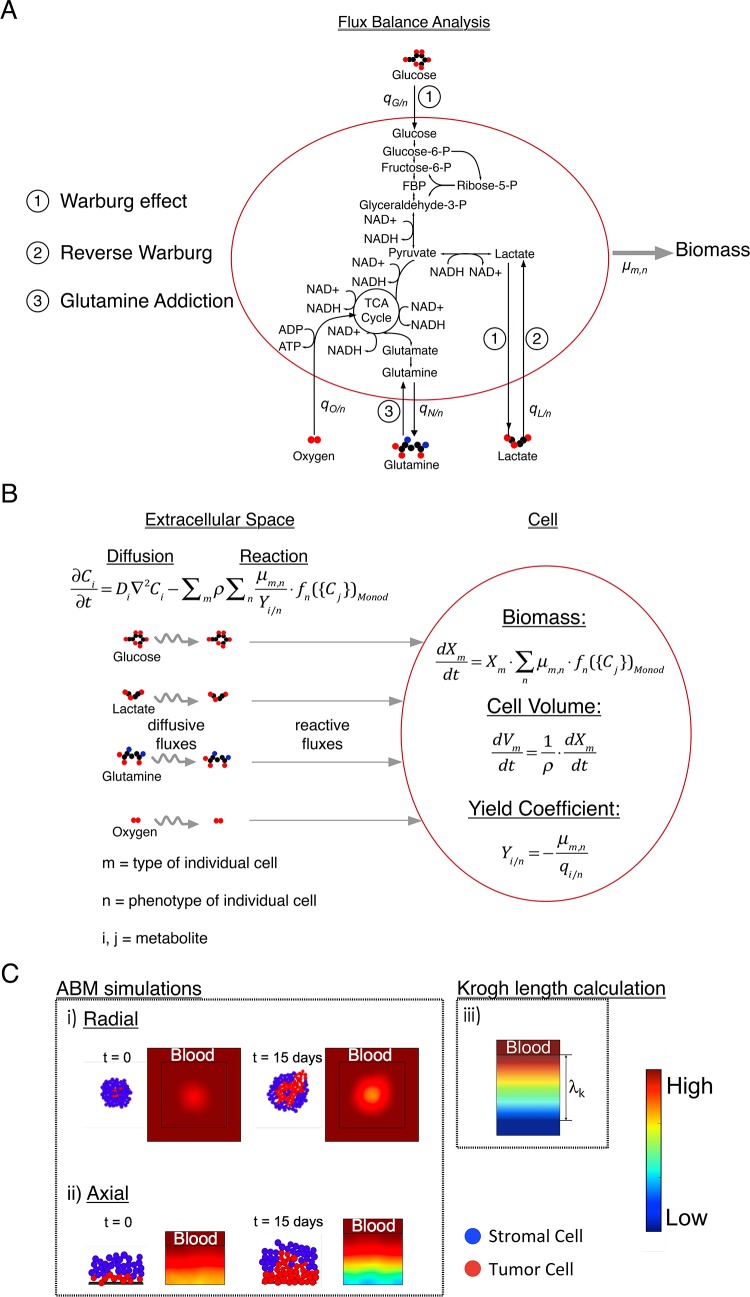
Multi-scale modeling of cancer metabolism. (A) Flux Balance Analysis (FBA). The arrows represent fluxes of species within a reduced representation of cell metabolism and cell growth; the detailed network used in FBA is presented in S1 Fig. Key steps associated with three hypotheses are labeled: ① Warburg effect, ② Reverse Warburg and ③ Glutamine addiction. The uptake and production rates are *q*_i/n_ [g/g-DW-hr] for the i^th^ metabolite and the n^th^ metabolic phenotype. We impose the maximum growth rate, *μ*_*m*,*n*_ [hr^-1^], for a given metabolic phenotype of each cell type as an objective function within the FBA. (B) Cell: Biomass (*X*_*m*_ [g]) growth of each cell type is modeled as a Monod-like process parameterized by the same maximum growth rates used in FBA that are modulated by functions of metabolite concentrations, *f*_*n*_({*C*_*j*_})_*Monod*_. The change in Volume of the cell (*V*_*m*_ [L]) is calculated from biomass growth by applying a constant density of the cell (*ρ*[g-DW/L]). Yield coefficients (*Y*_*i/n*_ [g-DW/g]) for each metabolite (*i*) and corresponding metabolic phenotype (*n*) are defined in terms of the uptake and production rates (*q*_*i/n*_) obtained from FBA. Extracellular space: Species balances for each explicit metabolite follow reaction-diffusion kinetics and govern the concentration profiles of metabolite at the multicellular scale. These equations (Eqs 1–4 in text) are integrated into and solved within an agent-based model (ABM—iDynoMiCS). (C) ABM simulations: i) Radial, two-dimensional growth: Tumor cells grow radially out from an initial cluster of cells with metabolites supplied at the edge of the cell mass such that radial gradients of concentration emerge (color map–red is high and blue is low concentration). Two phenotypes are displayed (red: tumor cells and blue: stromal cells). As the tumor grows, concentration gradients of metabolites become significant, making the tumor growth a diffusion-limited process that can result in different growth dynamics as well as distinct spatial distribution of cell subpopulations. ii) Axial, one-dimensional growth: Layers of tumor cells (red) and stromal cells (blue) are initiated near a blood vessel that supplies metabolites (from the top), such as glucose and oxygen in the blood stream. Growth pushes cells deeper into the tissue, away from the vessel, such that strong gradients of metabolite can again occur. iii) Krogh length calculation: To evaluate the impact of diffusion limitations in a simple model, we treat cells as continuum with uniform, zero^th^ order kinetics of metabolite consumption to calculate the distance over which the concentration of limiting metabolites falls to zero within the tumor mass; we refer to this distance as the Krogh length of a given metabolite.

**Fig 2:**
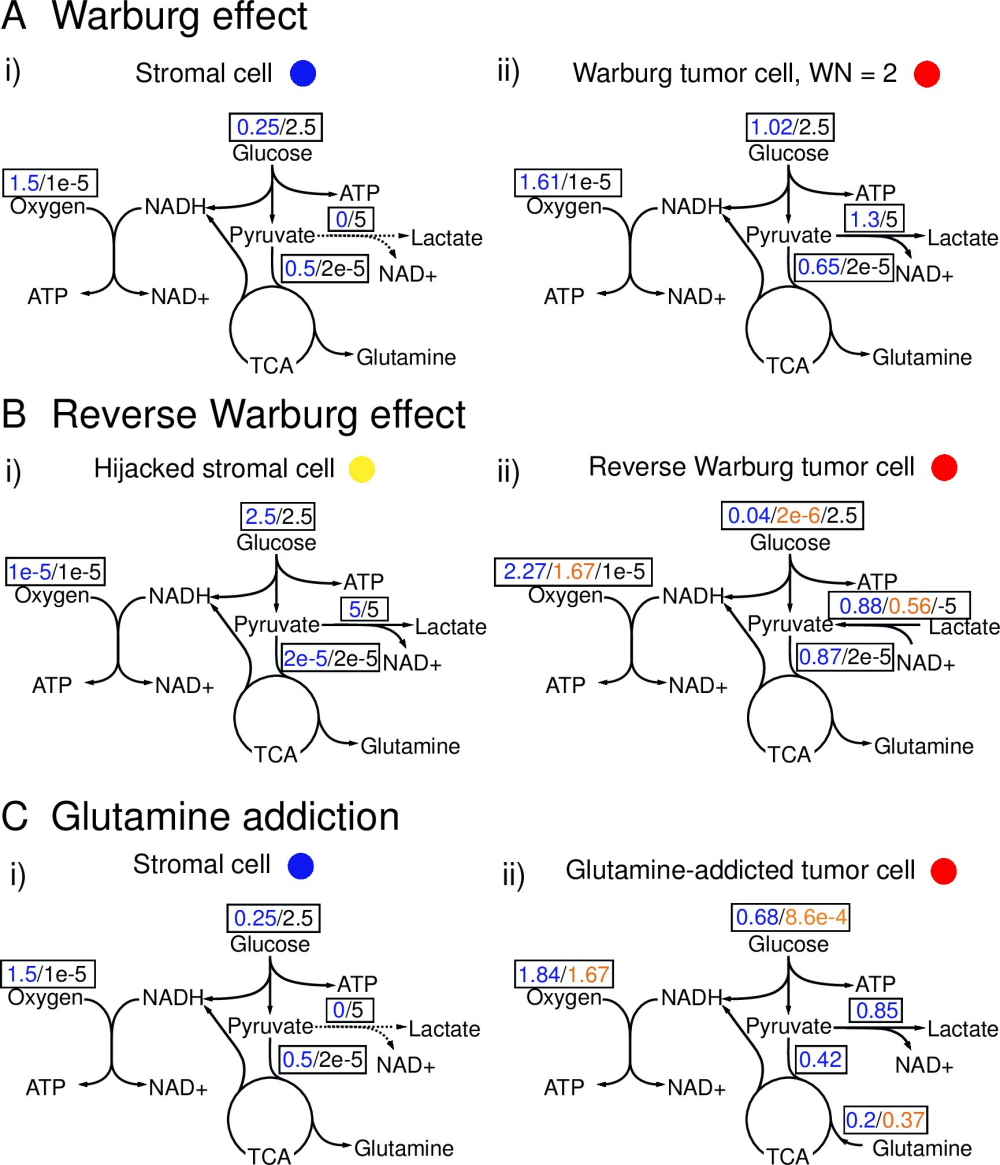
Metabolic profiles of various cell types in different hypotheses. Values of metabolic fluxes [mmol/g-DW-hr] under different metabolic phenotypes obtained from the FBA are shown in boxes. Blue: normoxic phenotype. Orange: hypoglycemic phenotype. Black: hypoxic phenotype. (A) Representation of the Warburg Effect hypothesis includes: (i) Stromal cell: Normoxic stromal cells are quiescent and aerobic (use mainly OXPHOS to generate ATP for maintenance) (blue values). Hypoxic stromal cells are quiescent and anaerobic (use primarily glycolysis to generate ATP for maintenance) (black values). (ii) Warburg tumor cell: Normoxic Warburg tumor cells are highly proliferative and aerobically glycolytic (use mainly glycolysis to generate ATP to grow—blue values). The values of flux shown represent the metabolic phenotype of Warburg Number, WN = 2. Hypoxic Warburg tumor cells are quiescent and anaerobically glycolytic (use glycolysis to generate ATP for maintenance—black values). (B) Representation of the Reverse Warburg effect includes: (i) Hijacked stromal cell: Normoxic hijacked stromal cells are quiescent and undergo aerobic glycolysis (use mainly glycolysis to generate ATP for maintenance—blue values). Hypoxic hijacked stromal cells are quiescent and anaerobic (use mainly glycolysis to generate ATP for maintenance—black values). (ii) Reverse Warburg tumor cell: Normoxic reverse Warburg tumor cells are highly proliferative and uptake lactate aerobically; however they utilize OXPHOS to generate ATP to grow, fueled by lactate and oxygen instead of undergoing glycolysis using glucose (blue values). Hypoglycemic reverse Warburg tumor cells are quiescent and consume lactate to fuel mitochondria for maintenance (orange values). Hypoxic reverse Warburg tumor cells are quiescent and undergo anaerobic glycolysis to produce lactate (black values). Note the different directions of arrows for lactate fluxes. (C) Representation of Glutamine Addiction includes: (i) Stromal cell: Normoxic stromal cells are quiescent and aerobic (use mainly OXPHOS to generate ATP for maintenance—blue values). Hypoxic stromal cells are quiescent and anaerobic (use primarily glycolysis to generate ATP for maintenance—black values. (ii) “Glutamine-addicted” tumor cell: Normoxic “glutamine-addicted” tumor cells are highly proliferative and aerobic; instead of utilizing glucose in glycolysis, they undergo OXPHOS to generate ATP to grow, fueled by glutamine and oxygen (blue values). Hypoglycemic “glutamine-addicted” tumor cells are quiescent and consume glutamine to fuel mitochondria for maintenance (orange values). Hypoxic “glutamine-addicted” tumor cells do not consume glutamine. They are quiescent and undergo anaerobic glycolysis (black values). Note the different directions of arrows for glutamine flux.

**Fig 3:**
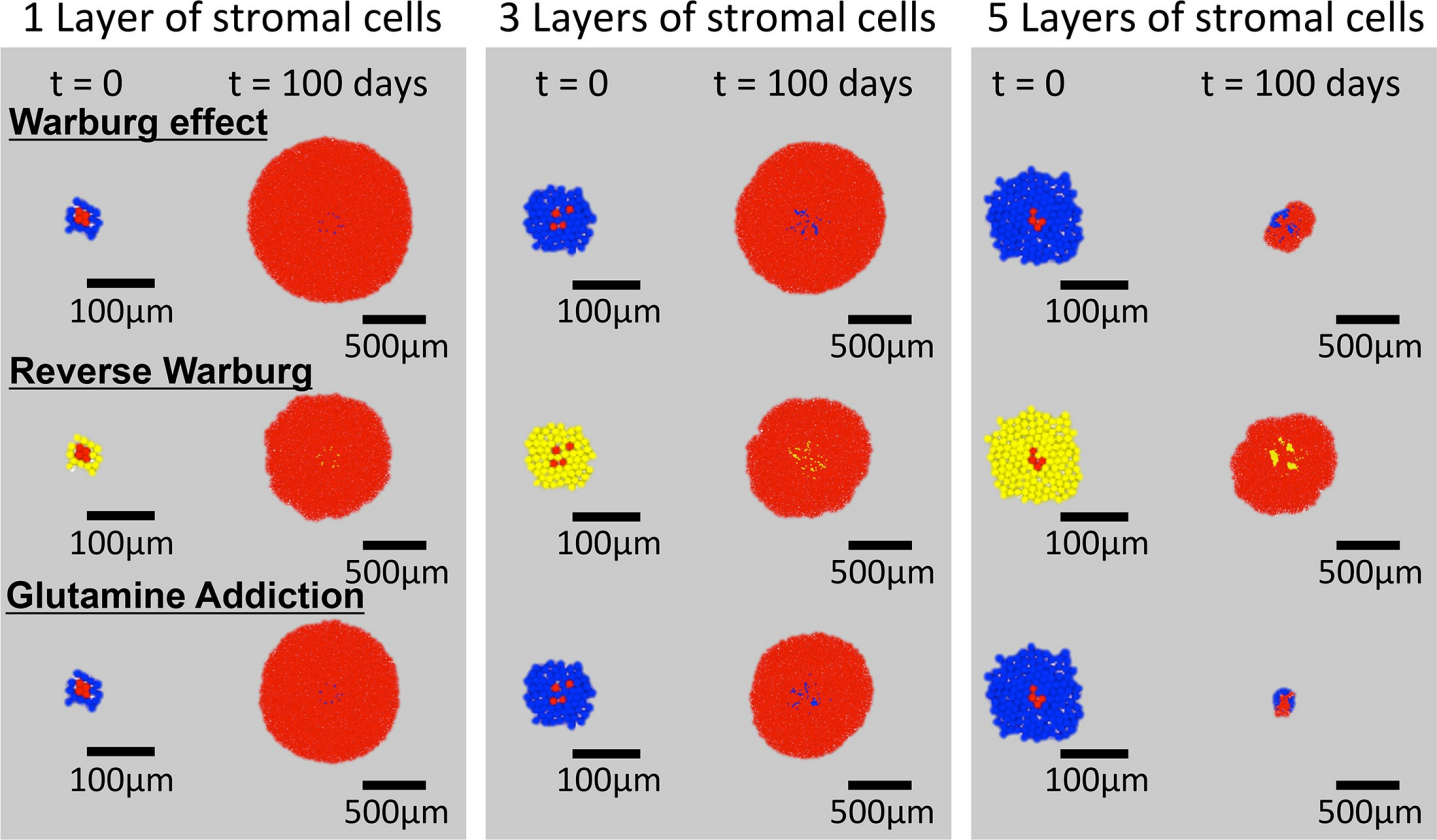
Distribution of cells in radial simulations. Initial conditions (t = 0) and end points (t = 100 days) are shown for the three hypotheses with cells seeded initially with 1, 3 and 5 layers of stromal cells surrounding the tumor cells.

In his seminal work, Warburg noted the distinct metabolic profile of tumor cells with high glycolytic rate and lactate production in the presence of oxygen. This so-called Warburg effect or aerobic glycolysis has been widely observed in different types of tumor cells (Fig 1A, ①) [8]. This original observation by Warburg led him to hypothesize that aerobic glycolysis is caused by impaired respiration; in turn, this defect results in cancer [9]. It is now well accepted that this hypothesis is incorrect as most tumor cells retain functional mitochondria [10,11]; we still lack a full understanding of the origin and consequences of the Warburg effect. More recently, other hypotheses have been proposed in the field of cancer metabolism such as the reverse Warburg effect (Fig 1A, ②) and glutamine addiction (Fig 1A, ③) [3,12–16]. Despite support of these three hypotheses from various experimental studies, significant uncertainty remains with respect to their definitions, their origin, and their impact on tumor progression and therapeutic interventions. Unraveling these fundamental questions could open a clearer path to targeting cancer metabolism as a therapeutic strategy.

In the past few years, studies of cancer metabolism have begun to elucidate how the metabolic alterations in tumor cells can influence tumor progression [9,17–21]. A definitive characteristic of tumor cells is uncontrolled proliferation. Compared to healthy cells that remain quiescent in most of their life cycle, tumor cells proliferate rapidly, accompanied by high rates of metabolic uptake. This metabolic profile of tumor cells leads to significant depletion of metabolites in the local microenvironment, resulting in resource limitations. Additionally, byproducts and waste products produced by the metabolism of tumor cells can potentially hinder the growth of neighboring cells or act as sources of alternative metabolic substrates [16,22,23]. Although studies have made efforts to capture these experimental observations mathematically [20,24–29], we are unaware of computational studies that test the implications of these hypotheses with respect to metabolic behaviors at the individual cell level, intercellular interactions mediated by shared metabolic environment, and the collective behavior that together define fitness and growth potential of the tumor. Recent computational work has made progress toward capturing the multi-scale complexity of cancer. These studies investigated the effect of tumor microenvironmental factors, specifically molecular cues and metabolites, on tumor population dynamics and provided insights into the cooperative behaviors of tumor subpopulations [30–34]. Similar intraspecies competition or cooperation are often observed in microbial organisms and heavily studied from a population ecology perspective [35–37]. Theories and modeling tools are better developed in the microbial field due to the relatively convenient validation from experiments [38–40].

In this study, we take a multi-scale modeling approach to describe the intracellular, cellular, and multicellular behaviors of cells within a tumor (Fig 1). With this framework, we investigate the following hypotheses: Warburg Effect/Aerobic glycolysis (①), Reverse Warburg (②), and Glutamine Addiction (③). We begin by translating hypotheses from experimental studies into constraints and objectives within the FBA (Fig 1A). We proceed to use FBA to obtain the yield coefficients (Y = maximum growth rate/flux of metabolite) for use in Monod-like kinetics of cellular growth at the individual cell level (Fig 1B). Finally, we simulate the growth dynamics of these cells at the multicellular scale to elucidate the implications of these metabolic scenarios (Fig 1C). We address the impact of the metabolic phenotypes implied by current hypotheses on the growth dynamics of tumor cells in the resource-limited microenvironments that emerge after tumor initiation. This modeling framework opens a route to explore tissue-scale tumor dynamics with explicit account taken for these metabolic scenarios in an efficient manner.

## Model

### Scale-bridging model formulation

Fig 1 illustrates, schematically, the multi-scale approaches we use. At the intracellular scale, we use Flux Balance Analysis (FBA) to construct a network that captures the central metabolism of mammalian cells (Fig 1A). In Fig 1A, the arrows represent fluxes of species within a reduced representation of cell metabolism and cell growth; the detailed network used in FBA is presented in S1 Fig. Key steps associated with three hypotheses are labeled: Warburg effect (①) is distinguished by high glycolytic flux and lactate production; reverse Warburg (②) is distinguished by the uptake of lactate; and glutamine addiction (③) is distinguished by uptake of glutamine as a carbon source to feed TCA cycle. We build the biomass template reaction (S1 Fig) based on major precursors for biomass synthesis by reducing Shlomi and coworker’s genome scale biomass template [20]. We impose a cellular maintenance reaction with a baseline rate to define the required minimum metabolism of cells (see Methods). We modify constraints and objective functions within the FBA network to define the characteristics of the different hypotheses (labeled in Fig 1A). We estimate parameters based on literature (see S1 Table). We acknowledge that the altered metabolic phenotype of tumor cells may be due to prior genetic events that occurred in the cell, such as loss of tumor suppressors (e.g., p53) [41]. However, we only consider the metabolic phenotypes of the cells at fixed genetic profiles here since we focus on impact of metabolic profiles on tumor growth over time scales (days) that are short relative to those required for the emergence and accumulation of genetic alterations in the cells (months or years). At the cellular scale (Fig 1B), we use the imposed maximum growth rates (*μ*_*m*,*n*_ [hr^-1^]) and the metabolic uptake and production rates of the metabolites (*q*_i/n_ [g/g-DW-hr]) obtained from FBA to determine yield coefficients ((*Y*_*i/n*_ [g-DW/g]) for each metabolite (*i*) and corresponding metabolic phenotype (*n*):

$$Y_{i/n}=-\frac{\mu_{m,n}}{q_{i/n}}$$ (1)

These yield coefficients link our intracellular treatment of metabolism by FBA and our cellular and multicellular treatment of resource utilization and growth. We model biomass (*X*_*m*_ [g]) growth of each cell type as a Monod-like process parameterized by maximum growth rate for each metabolic phenotype, *μ*_*m*,*n*_ [hr^-1^] and a Monod function of metabolite concentrations, *f*_*n*_({*C*_*j*_})_*Monod*_:

$$\frac{dX_{m}}{dt}=X_{m}\cdot \sum_{n}\mu_{m,n}\cdot f_{n}{\left(\left\{C_{j}\right\}\right)}_{Monod}$$ (2)

We provide detailed discussions of the Monod functions in the next **Section**. We used the same value of maximum growth rate for each phenotype of each cell at both the FBA (Fig 1A) and cell-scale (Fig 1B). We report parameter values in S1 Table. Additionally, we use a threshold in cell diameter to define the doubling of the cell by linking biomass growth to the volume (*V*_*m*_ [L]) expansion of the cell at a fixed dry mass density (ρ [g-DW/L]):

$$\frac{dV_{m}}{dt}=\frac{1}{\rho }\frac{dX_{m}}{dt}$$ (3)

To bridge the treatment of metabolic processes at the cellular and multicellular scales, we solve steady state species balances for each explicit metabolite at each time step within iDynoMiCS [39]:

$$\frac{\partial C_{i}}{\partial t}=D_{i}\nabla^{2}C_{i}+\sum_{m}\rho \sum_{n}q_{i/n}\cdot f_{n}{\left(\left\{C_{j}\right\}\right)}_{Monod}$$ (4)

where *C*_*i*_ [g/L] is the concentration of i^th^ metabolite, *D*_*i*_ [m^2^/day] is the diffusion coefficient of i^th^ metabolite. Here, the species balances can be safely treated as being at steady state because the time step in our simulation (1 hour) is selected to resolve cell growth and is long compared to typical transients in metabolism [39].

We integrate Eqs 1–4 into iDynoMiCS to track the growth of individual cells within a continuum matrix occupied by other cells in which metabolites diffuse (term 1 in Eq 4). The concentration of metabolites at the multicellular scale governs the cellular biomass growth (Eq 2) and the biomass kinetics in turn influences the concentration profile of metabolites (term 2 in Eq 4), and subsequently the growth kinetics of the surrounding cells. Cells are treated as hard spheres [38]. This spatial-temporal interaction between the cells and the microenvironment is a dynamic process that changes at each time step within iDynoMiCS.

We simulate growth in both radial and axial geometries in iDynoMiCS (Fig 1C): 1) Radial, two-dimensional growth (Fig 1C i))–tumor cells (red) grow radially out from an initial cluster of cells with metabolites supplied at the edge of the cell mass such that radial gradients of concentration emerge (color map). As the tumor grows, concentration gradients of metabolites become significant, making the tumor growth a diffusion-limited process that can result in different growth dynamics as well as distinct spatial distribution of cell subpopulations. 2) Axial, one-dimensional growth (Fig 1C ii))–layers of tumor cells (red) and stromal cells (blue) are initiated near a blood vessel that supplies metabolites (from the top), such as glucose and oxygen in the blood stream. Growth pushes cells deeper into the tissue, away from the vessel, such that strong gradients of metabolite can again occur.

The radial simulations (Fig 1C i)) provide a qualitative understanding of the growth dynamics in different metabolic scenarios; axial simulations (Fig 1C ii)) allow us to further quantify the observed dynamics. In both cases, we initiate tumor cell clones (same Monod parameters) surrounded by a varying number of layers of stromal cells (defined by distinct metabolic and growth parameters–see Fig 2 and Table 1). These arrangements capture tumor growth with initiation occurring at different distances from local vascular structure and thus at different levels of metabolic stress. We proceed to track growth as a function of depth of initiation and metabolic phenotype. We perform 11 replicates, with randomly seeded initial positions of tumor and stromal cells within their compartments; all other parameters in the simulation were kept the same across these replicates for each metabolic scenario. Additionally, we kept these random initial seeding positions of cells the same across simulations of the three metabolic scenarios to eliminate any effect that comes from the difference in the initial seeding when comparing the scenarios. As we are interested in initial stages of avascular growth, we do not account for later stage processes such as angiogenesis. Further, we do not account for cell death explicitly in our simulations; tumor cells in zones with severely depleted metabolites remain quiescent based on the Monod-like growth kinetics. When evaluating total tumor size, this assumption is equivalent to counting dead cell mass within the necrotic core as part of the tumor; this definition is consistent with that of previous studies [42–46].

**Table 1:** Summary of uptake/production rates and yield coefficients of metabolites under different cellular metabolic phenotypes.

	Cell Type	Phenotype	μ_m,n_	q_Glu/n_	q_O2/n_	q_Lac/n_	q_Gln/n_	Y_Glu/n_	Y_O2/n_	Y_Lac/n_	Y_Gln/n_
	(m)	(n)	(hr^-1^)	(g/g-DW-hr)	(g/g-DW-hr)	(g/g-DW-hr)	(g/g-DW-hr)	(g-DW/g)	(g-DW/g)	(g-DW/g)	(g-DW/g)
Warburg Effect	Healthy Stromal Cell	Aerobic	1×10^−6^	-0.045	-0.048	N/A	N/A	2.22×10^−5^	2.08×10^−5^	N/A	N/A
		Anaerobic	1×10^−6^	-0.45	-3.2×10^−7^	N/A	N/A	2.22×10^−6^	3.13	N/A	N/A
	Warburg Tumor Cell	Aerobic, WN = 0	0.018	-0.078	-0.064	N/A	N/A	0.231	0.281	N/A	N/A
		Aerobic, WN = 2	0.018	-0.183	-0.052	N/A	N/A	0.098	0.349	N/A	N/A
		Aerobic, WN = 10	0.018	-0.394	-0.0266	N/A	N/A	0.0456	0.68	N/A	N/A
		Aerobic, WN = 34	0.018	-0.573	-0.006	N/A	N/A	0.031	2.953	N/A	N/A
		Anaerobic	1×10^−6^	-0.45	-3.2×10^−7^	N/A	N/A	2.22×10^−6^	3.13	N/A	N/A
Reverse Warburg Effect	Hijacked Stromal Cell	Aerobic	1×10^−6^	-0.45	-3.2×10^−7^	0.45	N/A	2.22×10^−6^	3.13	-2.22×10^−6^	N/A
		Anaerobic	1×10^−6^	-0.45	-3.2×10^−7^	0.45	N/A	2.22×10^−6^	3.13	-2.22×10^−6^	N/A
	Reverse Warburg Tumor Cell	Aerobic, WN = 2	0.018	-0.183	-0.052	0.117	N/A	0.098	0.349	-0.154	N/A
		Aerobic, RW	0.018	-6.72×10^−3^	-0.073	-0.079	N/A	2.68	0.247	0.227	N/A
		Hypoglycemic	1×10^−6^	-3.73×10^−7^	-0.053	-0.05	N/A	2.68	1.87×10^−5^	2×10^−5^	N/A
		Anaerobic	1×10^−6^	-0.45	-3.2×10^−7^	0.45	N/A	2.22×10^−6^	3.13	-2.22×10^−6^	N/A
Glutamine Addiction	Healthy Stromal Cell	Aerobic	1×10^−6^	-0.045	-0.048	N/A	N/A	2.22×10^−5^	2.08×10^−5^	N/A	N/A
		Anaerobic	1×10^−6^	-0.45	-3.2×10^−7^	N/A	N/A	2.22×10^−6^	3.13	N/A	N/A
	Glutamine-addicted Tumor Cell	Aerobic	0.018	-0.122	-0.059	N/A	-0.03	0.148	0.305	N/A	0.607
		Anaerobic	1×10^−6^	-1.55×10^−4^	-0.053	N/A	-0.054	6.46×10^−3^	1.88×10^−5^	N/A	1.86×10^−5^

With the aim of providing intuition on the outcomes of simulations and characteristic physical parameters, we also calculate the Krogh length, shown schematically in Fig 1C iii). Here, we define the Krogh length of a metabolite as the length at which the concentration of metabolite becomes zero given the uptake of the metabolite with zero^th^ order growth kinetics for the cell phenotype in the region (see Methods). While this is an extremely simple model that couples zero^th^ order kinetics with a continuum description of reaction and diffusion in the tissue, we will show that it provides insights into the characteristics by which reaction and diffusion govern the growth of tumors.

With this multi-scale computational framework, we study the tumor population dynamics in a spatial-temporal manner and investigate the consequences of different hypotheses in cancer metabolism from a population ecology perspective. This perspective examines the impacts of phenotypic composition, spatial structure and reaction-diffusion on tumor growth.

### Distinct metabolic profiles of various cell types implied by metabolic scenarios

Before we further specify the hypotheses depicted in Fig 1 individually, we define the metabolic phenotypes of the cell types implicated in these hypotheses based on observations in the literature. We integrate our interpretations of these metabolic mechanisms into FBA to obtain the uptake and production rates of metabolites (see Table 1): In our approach, we assume each cell type (e.g., healthy stromal cell) can adopt more than one metabolic phenotype (e.g., aerobic under normoxic conditions and anaerobic under hypoxic conditions). These different metabolic phenotypes are implemented as objective functions and constraints in FBA and in turn, result in different flux distributions (Fig 2, coded by color). We then obtain yield coefficients (*Y*_*i/n*_) for the i^th^ metabolite in the n^th^ metabolic phenotype of cells by linking maximum growth rate (*μ*_*m*,*n*_) of the m^th^ cell type to the uptake and production rates (*q*_*i/n*_) (Eq 1); the Y_i/n_ serve as measures of the efficiency with which the metabolites generate biomass: the bigger the value of Y_i/n_ is, the more efficiently the n^th^ metabolic phenotype utilizes the i^th^ metabolite to grow.

Fig 2 summarizes predictions from FBA for the metabolic profiles of these cell types under distinct metabolic phenotypes. The metabolic switch from normoxia to hypoxia or to hypoglycemia leads to drastic changes in metabolic fluxes; the values in box represent fluxes of the specific metabolites when they display different metabolic profiles, coded by color (see caption). These flux distributions in turn lead to different uses of metabolites as reflected in yield coefficients (presented in Table 1). We present detailed description of each phenotype of the cells in the following subsections.

#### The Warburg effect

In our exploration of the Warburg effect, we use healthy quiescent cells to define the tissue that hosts the tumor cells (Fig 2A i) and 2C i)). We refer to these cells as stromal cells. This metabolic scenario imposes a direct competition for resources between the two sub-populations (stromal cells and tumor cells).

To define the metabolic character of stromal cells under normoxia at the intracellular scale with FBA, we set an objective function that targets an extremely low growth rate (1×10^−6^ hr^-1^, equivalent to a doubling time of 28881 days) to represent the quiescent nature of healthy somatic cells (Fig 2A i), blue). Additionally, we put unconstrained bounds (0 to ∞) on all fluxes in the network.

To define the stromal metabolic phenotype at the cellular scale, we expressed the growth kinetics in terms with oxygen and glucose as the limiting metabolites. We chose a Monod form that captures the Pasteur effect:

$$\frac{dX_{S}}{dt}=\left(\mu_{S,aer}\frac{C_{G}}{K_{G}+C_{G}}\frac{C_{O}}{K_{O}+C_{O}}+\mu_{S,ana}\frac{C_{G}}{K_{G}+C_{G}}\frac{K_{O}}{K_{O}+C_{O}}\right)X_{S}$$ (5)

where *X*_*S*_ [g] is the biomass of the stromal cell, *μ*_*S*,*aer*_ [hr^-1^] is the maximum growth rate under normoxia, *μ*_*S*,*ana*_ [hr^-1^] is the maximum growth rate under hypoxia, *C*_*G*_ [g/L] is the concentration of glucose, *K*_*G*_ [g/L] is the half saturation constant of glucose, *C*_*O*_ [g/L] is the concentration of oxygen, and *K*_*O*_ [g/L] is the half saturation constant of oxygen. The Monod form in Eq 5 approximates the behavior of a quiescent somatic cell whose growth is under regulatory control that follows Pasteur effect [47]: when oxygen concentration the cells experience is high (C_O_ >> K_O_), the first term on the right-hand side of Eq 5 dominates, simulating oxidative phosphorylation (OXPHOS); when oxygen concentration becomes low (C_O_ << K_O_), the second term (Eq 5, right-hand side) becomes dominant, capturing cells undergoing anaerobic glycolysis. We set the half saturation constants of the metabolites (except lactate, which is set to be the same as that of glucose) to be 1/10 of their physiological concentrations in blood circulation (See S1 Table) with the assumption that cells would experience phenotypic change when concentrations of the limiting metabolites drop by an order of magnitude. Since we use the same maximum growth rates under normoxia and hypoxia (*μ*_*S*,*aer*_ = *μ*_*S*,*ana*_ = 1×10^−6^ hr^-1^), Eq 5 can be further simplified to the following form:

$$\frac{dX_{S}}{dt}=\mu_{S}\frac{C_{G}}{K_{G}+C_{G}}X_{S}$$ (6)

Although this form indicates that the growth of stromal cells only depends on glucose availability, driven by the imposed weak growth rate, the FBA accounts for the demand for cellular maintenance under different cellular phenotypes (aerobic vs. anaerobic) such that the predicted yield coefficients for both oxygen and glucose depend strongly on local concentration of oxygen (Table 1). The different utilizations of metabolites (represented by yield coefficients) under the influence of oxygen availability in turn impact local concentrations of both oxygen and glucose, and thus lead to different growth rates of cells.

To define the character of a Warburg tumor cells under normoxia (Fig 2A ii), blue), we used the ratio of the flux of pyruvate to lactate to its flux into the mitochondrion; we call this ratio the Warburg number (WN). A literature survey suggested that a typical value of the WN in tumor cells is 2 (two pyruvates go to lactate for every one that enters the TCA cycle), though significant uncertainty remains and WN may be as large as ~ 10 [15,48,49]. In this study, we explore a range of WN in our simulations, from 0 to 34.

To define the Warburg phenotype at the intracellular scale in FBA, we chose the objective function to achieve a growth rate of 0.018 hr^-1^ (doubling time = 38 hrs), a typical doubling rate for cancer cells [48]. We then iteratively changed the uptake rate of oxygen to achieve a desired WN. This iterative process was done by setting the upper bound of the constraint on the oxygen uptake rate to be the same as that of the unconstrained FBA solution (the case of WN = 0) and then lowering it in each iteration until the desired WN was reached. This constraint on oxygen forced the uptake of more glucose and led to production of lactate (① in Fig 1A, blue in Fig 2A ii)). Without this imposed constraint, our flux distribution did not display the characteristics of the Warburg effect (i.e., there was no lactate production such that WN = 0); an observation also made previously [20,50]. We found that a constraint directly imposed on lactate production could also be used to produce the same flux distribution predicted with constrained oxygen uptake. The equivalence of these two constraints is due to the requirement of ATP and redox balance to meet the growth demand; this balance can only be achieved via either OXPHOS or aerobic glycolysis [9,11]. As illustrated in Fig 2A ii) (WN = 2), when a Warburg phenotype is imposed (WN >0), the metabolic behavior of tumor cells under normoxia (blue) is very distinct from the Pasteur behavior of the healthy stromal cells (Fig 2A i)), as tumor cells undergo aerobic glycolysis. The Warburg phenotype under normoxia forces tumor cells to use glycolysis in addition to OXPHOS for ATP generation; this situation leads to a shift in utilization from oxygen to glucose, reflected in uptake rates of glucose (q_Glu/aer_ = -0.078 g/g-DW-hr for WN = 0 vs. q_Glu/aer_ = -0.183 g/g-DW-hr for WN = 2) as well, shown in Table 1.

To define the Warburg phenotype at the cellular scale, we selected a Monod form of growth kinetics that captures a Pasteur-like switch from rapid growth in normoxic conditions (aerobic growth, *μ*_*W*,*aer*_ = 0.018 hr^-1^) to slow growth under hypoxic conditions (anaerobic growth, *μ*_*W*,*ana*_ = 1×10^−6^ hr^-1^), depending on the local oxygen concentration:

$$\frac{dX_{W}}{dt}=\left(\mu_{W,aer}\frac{C_{G}}{K_{G}+C_{G}}\frac{C_{O}}{K_{O}+C_{O}}+\mu_{W,ana}\frac{C_{G}}{K_{G}+C_{G}}\frac{K_{O}}{K_{O}+C_{O}}\right)X_{w}$$ (7)

where *X*_*W*_ [g] is the biomass of the tumor cell, *C*_*G*_ [g/L] is the concentration of glucose, *K*_*G*_ [g/L] is the half saturation constant of glucose, *C*_*O*_ [g/L] is the concentration of oxygen, and *K*_*O*_ [g/L] is the half saturation constant of oxygen (S1 Table). When oxygen concentration the cells experience is high (C_O_ >> K_O_), the first term on the right-hand side of Eq 7 dominates, Warburg tumor cells adopt more of an aerobic, rapid growth profile (aerobic glycolysis); when oxygen concentration becomes low (C_O_ << K_O_), the second term (Eq 7, right-hand side) becomes dominant and tumor cells undergo much slower growth regime via anaerobic glycolysis.

#### The reverse Warburg effect

In the reverse Warburg hypothesis, oxidative tumor cells have been observed to uptake lactate as a carbon source in addition to glucose (Fig 1A, ②) [22,23,51]. Additionally, upregulation of glycolytic enzymes such as PKM2 has been observed in tumor-associated fibroblast suggesting an aerobic glycolytic phenotype for tumor-associated stromal cells [12,52]. This metabolic scenario represents an example of a host-parasite effect in which the hijacked stromal cells (the “host”) are feeding the tumor cells (the “parasite”) lactate by adopting an aerobic glycolytic phenotype [53]. This type of behavior between the oxidative and hypoxic tumor cells as well as between the tumor cells and the stromal cells has also been previously referred to as a symbiosis [14,16,24,25,29,54].

In the exploration of the reverse Warburg hypothesis, we used the “hijacked” stromal cells described by Sotgia *et al*. to define the tissue in which tumor grows [55]. These metabolically reprogrammed stromal cells can be tumor-associated fibroblasts or macrophages. Unlike the quiescent healthy stromal cells that undergo the Pasteur effect, they commit to a glycolytic phenotype in which they uptake glucose and produce lactate under both normoxic (blue in Fig 2B i)) and hypoxic (black in Fig 2B i)) conditions.

To capture the metabolic phenotype of aerobic glycolysis in hijacked stromal cells under normoxic conditions in FBA, we used an objective function to minimize oxygen uptake rate while constraining the cell at a low growth rate, *μ*_*HS*_ (1×10^−6^ hr^-1^, same as for healthy stromal cells). Hence, the metabolic flux distributions are identical in normoxic and hypoxic conditions, as shown in Fig 2B i).

We define the biomass growth of such reprogrammed stromal cells at the cellular scale as follows:

$$\frac{dX_{HS}}{dt}=\mu_{HS}\frac{C_{G}}{K_{G}+C_{G}}X_{HS}$$ (8)

where *X*_*HS*_ [g] is the biomass of the hijacked stromal cell, *C*_*G*_ [g/L] is the concentration of glucose, and *K*_*G*_ [g/L] is the half saturation constant of glucose (S1 Table). As with healthy stromal cells, this Monod form is also independent of oxygen. However, in this case, we follow the proposal by Sotgia *et al*. that these reprogrammed stromal cells are committed to a glycolytic phenotype that favors the use of oxygen and lactate by the adjacent Reverse Warburg tumor cells. Therefore, the yield coefficients of oxygen and glucose for the hijacked stromal cells remain the same under both normoxia and hypoxia (Table 1).

To investigate the hypothesis of reverse Warburg effect, we define the normoxic reverse Warburg phenotype of tumor cells at the intracellular scale in FBA by using an objective function to minimize the uptake of glucose to simulate the ability to utilize lactate as the preferred substrate by the tumor cells while constraining the growth rate, *μ*_*RW*,*aer*_, to be high under normoxic conditions (0.018 hr^-1^ as with the Warburg phenotype) (blue in Fig 2B ii)).

At the cellular scale, based on our interpretation of the reverse Warburg tumor cells from the literature [12,16,23], we allowed them to adapt to different metabolic phenotypes in response to changes in local concentration of metabolites (i.e., lactate, glucose and oxygen), captured by the Monod-like growth kinetics. Specifically, in addition to the normoxic Warburg and hypoxic phenotypes (Fig 2A ii)), we introduced two more metabolic phenotypes, the normoxic reverse Warburg (blue in Fig 2B ii)) and hypoglycemic phenotypes (orange in Fig 2B ii)) to describe the reverse Warburg tumor cells:

$$\frac{dX_{RW}}{dt}=\mu_{RW,aer}\frac{C_{G}}{K_{G}+C_{G}}\frac{C_{L}}{K_{L}+C_{L}}\frac{C_{O}}{K_{O}+C_{O}}+\mu_{W,aer}\frac{C_{G}}{K_{G}+C_{G}}\frac{K_{L}}{K_{L}+C_{L}}\frac{C_{O}}{K_{O}+C_{O}}+\mu_{hypogly}\frac{C_{L}}{K_{L}+C_{L}}\frac{C_{O}}{K_{O}+C_{O}}\frac{K_{G}}{K_{G}+C_{G}}+\mu_{RW,ana}\frac{C_{G}}{K_{G}+C_{G}}\frac{K_{O}}{K_{O}+C_{O}})X_{RW}$$ (9)

where *X*_*RW*_ [g] is the biomass of the tumor cell, *μ*_*W*,*aer*_ [hr^-1^] is the maximum growth rate of the Warburg phenotype under normoxia, *μ*_*hypogly*_ [hr^-1^] is the maximum growth rate under hypoglycemia, and *μ*_*RW*,*ana*_ [hr^-1^] is the maximum growth rate under hypoxia; *C*_*G*_, *C*_*O*_, and *C*_*L*_ [g/L] are the concentrations of glucose, oxygen and lactate, and *K*_*G*_, *K*_*O*_ and *K*_*L*_ [g/L] are the half saturation constants of glucose, oxygen and lactate (S1 Table).

Eq 9 encodes the following characteristics of our interpretation of the reverse Warburg hypothesis: 1) when lactate is abundant (*C*_*L*_ >> *K*_*L*_), the tumor cells preferably uptake lactate over glucose and undergo OXPHOS aided by oxygen to grow under normoxia (blue in Fig 2B ii), normoxic reverse Warburg phenotype, term 1 in Eq 9, aerobic growth, *μ*_*RW*,*aer*_ = 0.018 hr^-1^). 2) When lactate is limited (*C*_*L*_ << *K*_*L*_), we allow the tumor cells under the hypothesis of reverse Warburg effect to revert back to the Warburg phenotype described above and grow by taking up glucose while producing lactate (term 2 in Eq 9, aerobic growth, *μ*_*W*,*aer*_ = 0.018 hr^-1^). We note that due to the equality in the maximum growth rates in both Reverse Warburg and Warburg phenotypes, term 1 and 2 in Eq 9 can be combined leading to an independence of lactate in the aerobic growth conditions. 3) When glucose is limiting, we allow the tumor cells to stay quiescent (orange in Fig 2B ii), hypoglycemic phenotype, term 3 in Eq 9, *μ*_*hypogly*_ = 1×10^−6^ hr^-1^) by having lactate and oxygen generate the energy necessary for cell maintenance. We achieve this hypoglycemic metabolic phenotype by imposing the same objective function and constraints as the reverse Warburg phenotype in FBA but at a growth rate, *μ*_*hypogly*_ = 1×10^−6^ hr^-1^ (see Methods). 4) The reverse Warburg tumor cells are also sensitive to local oxygen concentration. When oxygen becomes limiting, they utilize glucose in anaerobic fermentation to stay quiescent (black in Fig 2B ii), hypoxic phenotype, term 4 in Eq 9, *μ*_*RW*,*ana*_ = 1×10^−6^ hr^-1^), same as the Warburg tumor cell, and healthy stromal cells under hypoxic conditions (also see Methods).

#### Glutamine addiction

Glutamine addiction has emerged as one of the most acknowledged hypotheses in the field of cancer metabolism [6]: Although glutamine addiction is not observed in all tumor cells, the community has started to recognize the role of glutamine in growth as a hallmark of metabolic rewiring in cancer [6,13,56,57]. Numerous studies reported that tumor cells consume glutamine via glutaminolysis to feed their TCA cycle, a process termed anaplerosis (Fig 1A). In this study, we aim to explore specifically the role of glutamine in anaplerosis by considering glutamine as an alternative substrate for glucose-derived carbon in the mitochondria of tumor cells. Here, we make a simplified assumption that the growth of tumor cells is hindered under glucose deprivation due to the dependence on upstream glycolysis and the pentose phosphate pathway, capturing a coupled utilization of glucose and glutamine in cancer metabolism [56]. Following this assumption, we model glutamine-addicted tumor cells that cannot survive on glutamine as the sole carbon source, consistent with the behavior of MYC-positive tumor cells [58,59]. Since the Warburg effect and glutamine addiction are not mutually exclusive hypotheses [60], we created the glutamine-addicted tumor cells by adding the dependence of glutamine to the previously defined Warburg phenotype of tumor cells (with a WN of 2). Hence, glutamine-addicted tumor cells utilize both glucose and glutamine as non-equivalent carbon sources to grow, with glutamine used in anaplerosis only.

To define the glutamine-addicted phenotype of tumor cells, at the intracellular level with FBA, in addition to the constraints that results in a WN of 2, we also constrained the network such that the ratio of glutamine to glucose uptake rates was 3 to 10 as observed experimentally [49]. This specific constraint forces the FBA network to uptake glutamine as a carbon source that would not occur autonomously. Specifically, we set the growth rate to be 0.018 hr^-1^, increased the upper bound of the constraint on glucose while decreasing the upper bound of the constraint on oxygen and sought for a value for glutamine uptake by changing the upper bound of the constraint on glutamine that allowed the ratio of fluxes of glutamine to glucose to be 3 to 10 and WN = 2 (blue in Fig 2C ii)). As in the treatment of Reverse Warburg tumor cells, we allow the glutamine-addicted tumor cells to remain quiescent under hypoglycemic conditions by having both glutamine and oxygen fuel their mitochondria to generate the energy necessary for cell maintenance (orange in Fig 2C ii)). We achieve this hypoglycemic metabolic phenotype by setting the objective function to minimize glucose uptake rate while constraining the growth rate at 1×10^−6^ hr^-1^ as well as allowing uptake of glutamine (also see Methods). We note that due to the requirement of oxygen for the utilization of glutamine in energy production, growth of glutamine-addicted tumor cells under hypoxia depends on oxygen. Hence, we describe the growth kinetics of glutamine-addicted tumor cells at the cellular scale as follows:

$$\frac{dX_{GA}}{dt}=\left(\mu_{GA,aer}\frac{C_{G}}{K_{G}+C_{G}}\frac{C_{N}}{K_{N}+C_{N}}\frac{C_{O}}{K_{O}+C_{O}}+\mu_{hypogly}\frac{C_{N}}{K_{N}+C_{N}}\frac{C_{O}}{K_{O}+C_{O}}\frac{K_{G}}{K_{G}+C_{G}}\right)X_{GA}$$ (10)

where *N* refers to glutamine, *μ*_*GA*,*aer*_ [hr^-1^] is the maximum growth rate of the glutamine-addicted phenotype under normoxia, *μ*_*hypogly*_ [hr^-1^] is the maximum growth rate under hypoglycemic conditions, *C*_N_ [g/L] is the concentration of glutamine and *K*_*N*_ [g/L] is the half saturation constant of glutamine (S1 Table).

Again, we used healthy quiescent cells to define the tissue that hosts the tumor cells (Fig 2A i) and 2C i)).

## Results

### Radial simulations

To gain a qualitative understanding of the impact of the various metabolic scenarios on tumor growth in a diffusion-limited microenvironment, we first ran simulations in an unconstrained 2-D domain, as shown in Fig 1C i); metabolites were delivered through a diffusive boundary layer of fixed thickness that surrounds the growing tissue (see Methods). Fig 3 presents the form of the tumors at initiation (t = 0) and after 100 days of growth for Warburg tumor cells (WN = 2) with healthy stromal cells (top row), Reverse Warburg cells with hijacked stromal cells (middle row), and glutamine-addicted tumor cells with healthy stromal cells (bottom row). The three columns are for initial seeding of tumor cells beneath 1, 3, and 5 layers of stromal cells, as indicated in the images of the initial configuration of the cells (t = 0). As the number of layers of stromal cells increases, the growth of tumor cells becomes compromised due to the reduced access to the metabolites. By hindering diffusion and consuming oxygen and glucose, the stromal cells decrease the accessibility of these metabolites to the tumor cells. In all cases, we note that the proliferation of the tumor cells led to their breaking through the layers of stromal cells; for the cases with significant growth, the stromal cells became engulfed within the tumor, as is frequently observed in actual tumors [61]. We also note the emergence of irregular front of the tumor in the scenario of Reverse Warburg effect. We suspect that this irregularity arises from growth instability due to the moderate availability of metabolites at the growth front [35]. We note qualitatively different effects of the addition of layers of stromal cells on tumor growth for the different scenarios: with 5 layers of stromal cells, the growths of both Warburg and Glutamine-addicted tumor cells were strongly delayed, whereas the impact on the growth of Reverse Warburg cells was modest. These observations motivate a deeper investigation of the mechanisms that control response to metabolic stress in these scenarios.

### Impact of reaction-diffusion on tumor growth

**Fig 4:**
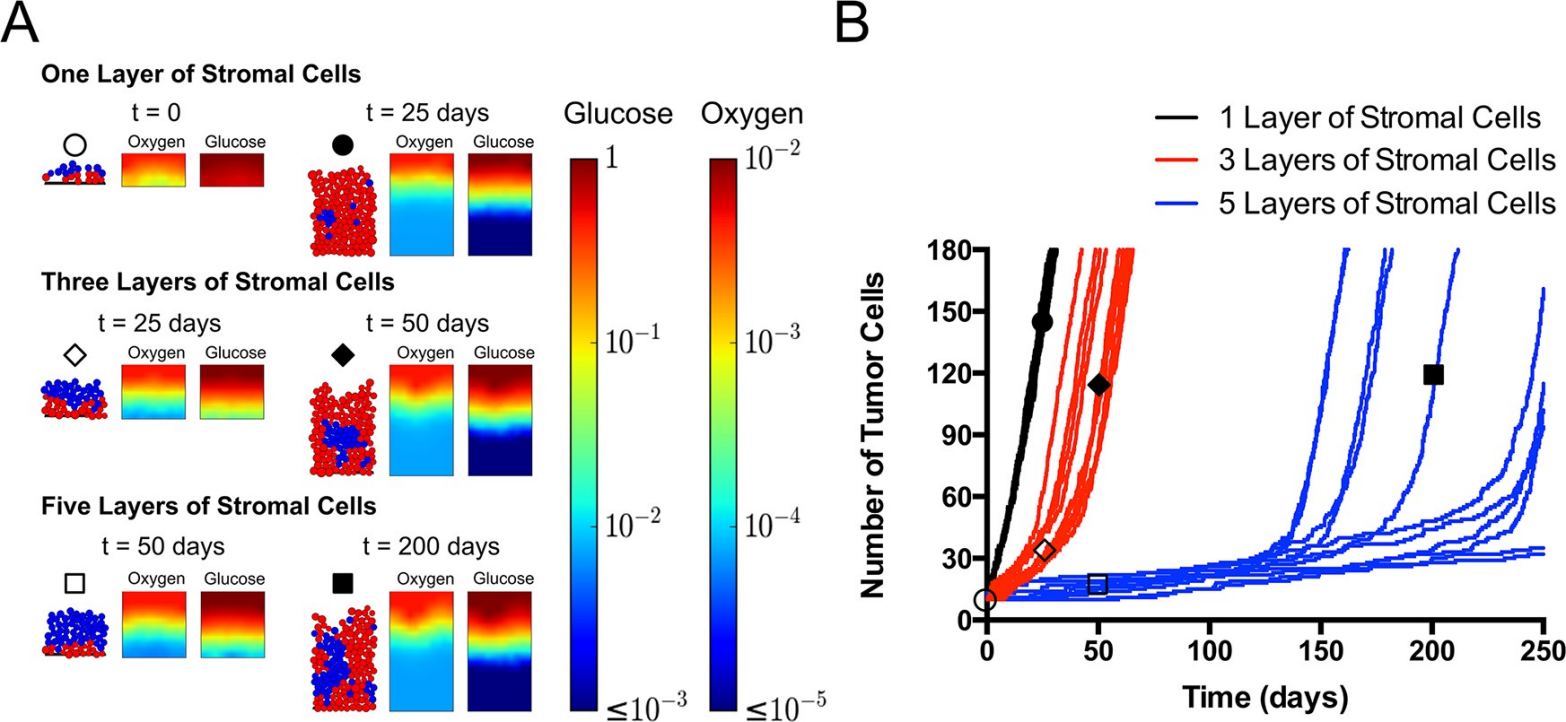
Axial agent-based model of growth of Warburg tumor cells in a perivascular tissue. (A) Snapshots of predicted cellular structure and concentration fields of metabolites at different times. Tumor cells with one, three, and five of layers of healthy stromal cells separating them from the source of nutrients (top, representing interface with blood). Color bars present concentrations of oxygen and glucose in g/L. (B) Growth trajectories of tumor cells from simulations in three cases in (A). For each case, the trajectories for 11 independent simulations are shown. Initial positions of cells were randomly generated within the corresponding stromal or tumor compartment (see Methods). Empty circle: one layer of stromal cells; time = 0 day. Filled circle: one layer of stromal cells, t = 25 days. Empty diamond: three layers of stromal cells; t = 25 days. Filled diamond: three layers of stromal cells; t = 50 days. Empty square: five layers of stromal cells; time = 50 days. Filled square: five layer of stromal cells; t = 200 days.

We proceeded to dissect the metabolic scenarios further with simulations in a confined geometry in which solute (i.e., metabolite) diffusion and tissue expansion were constrained along a single direction, as shown in Fig 1C ii). This axial scenario approximates the local environment adjacent to a blood vessel (upper boundary). Fig 4 presents an overview of the growth behavior in this geometry. For this overview, we simulated the Warburg scenario, with Warburg tumor cells (WN = 2) and healthy stromal cells (also see Fig 2).

Fig 4A shows the snapshots of tumor growth and the corresponding concentration fields of oxygen and glucose at various time-points for tumors initiated beneath 1, 3, and 5 layers of stromal cells. In the colormaps of the concentration fields, we see that when the tumor initiated closer to the source (top row with 1 layer of stromal cells), the Warburg tumor cells had access to ample oxygen and glucose to fuel their growth at early time (t = 0, empty circle); at late time (t = 25 days, filled circle), significant depletion of both oxygen and glucose occurred, but the uppermost layer of tumor cells still benefited from high metabolite concentrations to grow. However, when the tumor initiated farther away from the source (middle and bottom rows), the diffusion limitations and consumption by the stromal cells limited the metabolites available to the tumor cells, even at early times (empty diamond, empty square). This limitation persisted until the tumor cells broke through the stromal layer and gained access to higher concentrations of metabolites (filled diamond, filled square).

Fig 4B presents the trajectories of tumor growth from 11 simulation runs in each case shown in Fig 4A. We first note that for all initial conditions, the growth appears to proceed through two phases, starting with slower growth that then transitions to faster growth; these two regimes are most evident for 3 and 5 layers of stromal cells. By observing the cellular configurations in the simulations (see S1–S3 Movies), we identify that the transition occurs when the tumor cells break through the layers of stromal cells and gain access to high concentrations of metabolites. When the tumor cells started to grow, the reaction-diffusion in the intact layers of stromal cells limited the supply of metabolites to the tumor cells. Under such conditions, the growth of tumor cells was significantly compromised due to the lack of oxygen (note the more severe depletion of oxygen relative to glucose in Fig 4A, also see Eq 7 in Model); the microtumor was nearly quiescent. Once this slow growth led to the penetration of one or more tumor cells through the layers of stromal cells, those tumor cells transitioned toward their aerobic growth regime (term 1 in Eq 7) and quickly overwhelmed the stroma. Interestingly, the growth rates after breakthrough were constant (the growth curves are linear in time) and independent of initial conditions (all late-time slopes are the same in Fig 4B). This constant growth rate is distinct from the exponential growth that one would expect resulting from saturating Monod-like growth kinetics (Eq 5–10 in Model). This observation illustrates an important consequence of a diffusion limited microenvironment. We will comment further on the origin of this constant rate below.

In the case of 1 layer of stromal cells (black curves), the growth transitions rapidly (within the first days) to a high, constant rate. Furthermore, the trajectories of all the random initial seeding conditions are very similar. For 3 and 5 layers of stromal cells (blue and red curves), the first, slow phase lasts longer because the tumor cells experienced more severe limitations in their initial configurations. Additionally, in these cases, the trajectories of different initial conditions diverge strongly from one another due to the differences in the moment of transition from slow to fast growth. This observation reflects the fact that the time for tumor cells to break through the stroma is sensitive to small differences in the initial configuration of cells.

### Fitness conferred by metabolic scenarios

We now proceed to use axial simulations like those in Figs 1C ii) and 4 to investigate the growth dynamics in each of the three metabolic scenarios.

#### The Warburg effect

**Fig 5:**
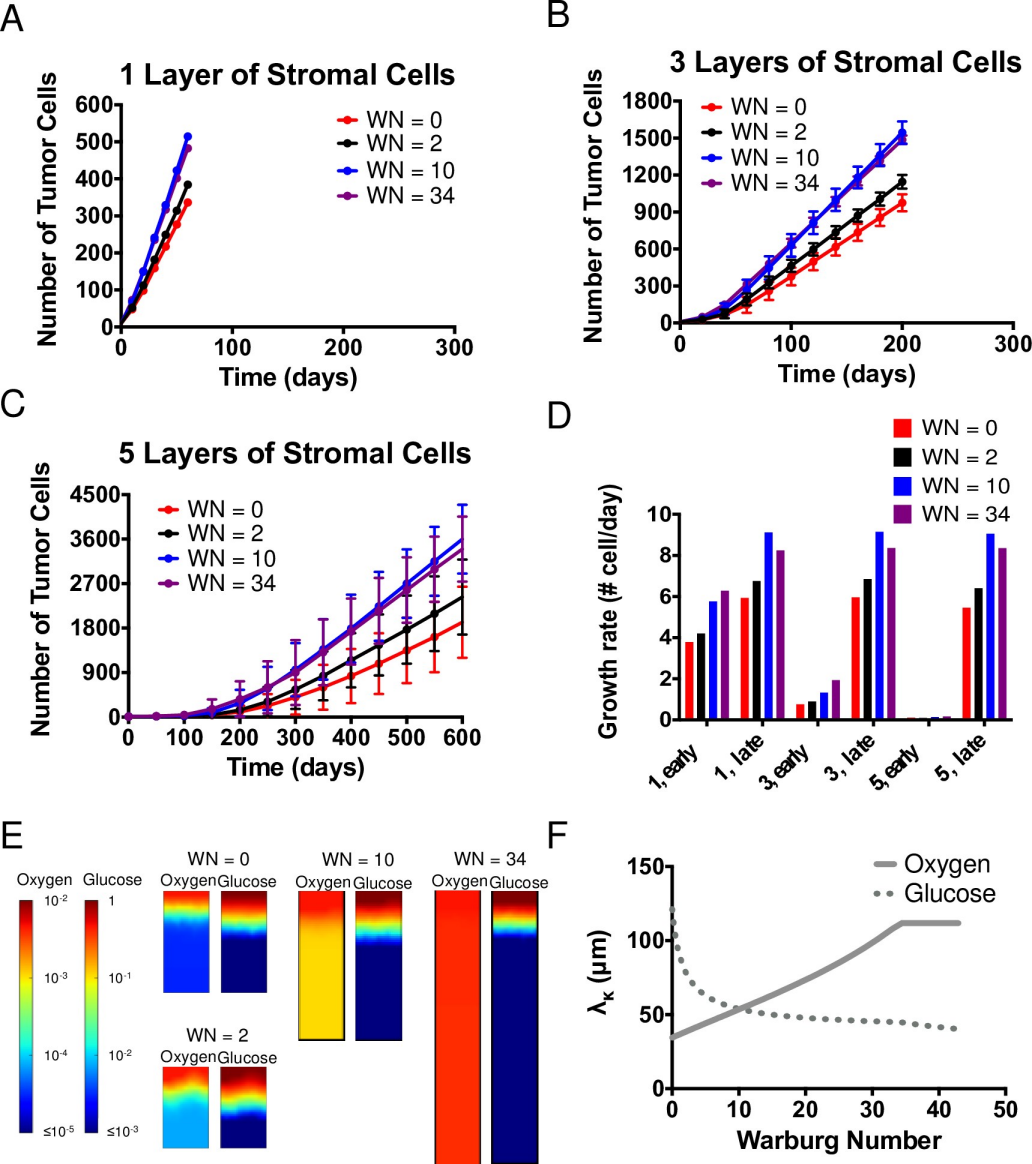
The Warburg effect. (A-C) Comparisons of growth curves from axial simulations as in Fig 4A for Warburg tumor cells with Warburg Number, WN = 0 (Control), 2, 10, and 34 with 1, 3 and 5 layers of stromal cells on top. Each time point represents the average of 11 simulations; error bars represent standard deviation. Note differences in vertical scales on plots. (D) Comparison of growth rate of tumor cells at early and late times (see Methods), extracted from the average growth curves in (A-C). (E) Concentration fields of metabolites in the case of 5 layers of stromal cells at *t* = 150 days. (F) Krogh lengths of oxygen and glucose based on consumption of tumor cells vs. Warburg Number. Solid line: Oxygen. Dotted line: Glucose.

In order to investigate the impact of the strength of Warburg effect on tumor growth in a resource-limited microenvironment, Fig 5 presents growth in the axial simulation (as in Fig 4) run with tumor cells that display various levels of the Warburg effect, as defined by the value of the Warburg Number (WN = 0, 2, 10, 34). Although all four different metabolic phenotypes of tumor cells grow at the same maximum growth rate under aerobic growth regime, due to different flux distributions of metabolites, tumor cells with higher WN have higher yield coefficients of oxygen and lower yield coefficients of glucose, as shown in Table 1.

Fig 5A–5C present the comparison of growth curves of tumor cells with three different thicknesses of stromal cells (Fig 4; circle: 1 layer of stromal cells; diamond: 3 layers of stromal cells; square: 5 layers of stromal cells). We first note that the two-phase growth regime is present for 3 and 5 layers of stromal cells across all four metabolic tumor phenotypes (Fig 5B and 5C). As before, the late-time rates are constant (linear growth curves). We note that the rate of late-stage growth increases with increasing WN up to WN = 10, before saturating or decreasing slightly (WN = 34).

To further quantify both early- and late-stage growths across these scenarios, Fig 5D presents growth rates extracted from the average curves in Fig 5A–5C (see Methods). In the cases of 1 and 3 layers of stromal cells at early times (Fig 5D, “1, early”, “3, early”), we observe that higher WN leads to faster growth. This observation suggests that when tumor cells experience moderate to high concentrations of metabolites near the blood vessels, the higher yields on oxygen at higher WNs provide a growth advantage. However, this trend is not obvious in the early-time growth rate of tumor cells in the case of 5 layers of stromal cells: the growth rates increase monotonically with WN, such that breakthrough occurs earlier for the more glycolytic cells (WN = 34, see Fig 4C). The late-time growth rates (Fig 5D, “late”) represent the cell growth after breaking through layers of stromal cells, when the outermost tumor cells have direct access to high concentrations of both oxygen and glucose regardless of the initial number of layers of stromal cells; these growth rates are a strong function of WN. Distinct from the early time behavior, the late-time growth rate of tumor cells is non-monotonic in WN: it rises from 0 to a maximum at WN = 10 before falling again at WN 34.

To understand this non-monotonic dependence on WN, Fig 5E presents the late-time concentration fields of metabolites for the case of 5 layers of stromal cells. These distributions show that the depletion of oxygen becomes significantly less severe as WN increases due to the increase in yield coefficients on oxygen (Table 1). More subtly, the depletion of glucose increases with increasing WN. We further calculated the Krogh lengths of metabolites based on tumor cell consumptions to provide insights into the predictions of growth rate from simulations at late times. Fig 5F shows the changes in Krogh lengths of oxygen (solid line) and glucose (dotted line) as WN increases. At low WN, the Krogh length of oxygen is smaller, indicating that the growth of tumor cells is mainly limited by the availability of oxygen. As WN increases, the Krogh length of oxygen increases (with increasing yield coefficient) whereas Krogh length of glucose decreases, and the two cross at WN ≅ 10. At higher WNs, growth is glucose-limited and the growth rate decreases with the decreasing Krogh length for glucose, as observed in the simulations (Fig 5D–“late”). Interestingly, this observation suggests that tumor cells may have optimal growth fitness at intermediate WN.

The observations of the late-time concentration fields in Fig 5E and the consideration of Krogh lengths allow us to explain the constant, late-time growth rates that we have noted above. The depletion of metabolites (oxygen and glucose) over a fixed distance within the growing tumor means that only cells within this peripheral zone (i.e., within a Krogh length of the source) grow while cells deeper within the tissue are essentially quiescent. A fixed number of cells growing at a constant rate lead to the constant growth of the tumor, in contrast to the more familiar scenario in which a homogeneous population grows exponentially with individual cell growing at a fixed rate. Our model thus captures and explains an important characteristic of solid tumor growth that has been observed experimentally [62,63].

#### The reverse Warburg effect

To study the population-scale effects of the reverse Warburg effect under resource limitations, we performed agent-based simulations by seeding hijacked stromal cells (Fig 2B i)) and the reverse Warburg tumor cells (Fig 2B ii)) in the axial geometry (Figs 1C ii) and 4A).

**Fig 6:**
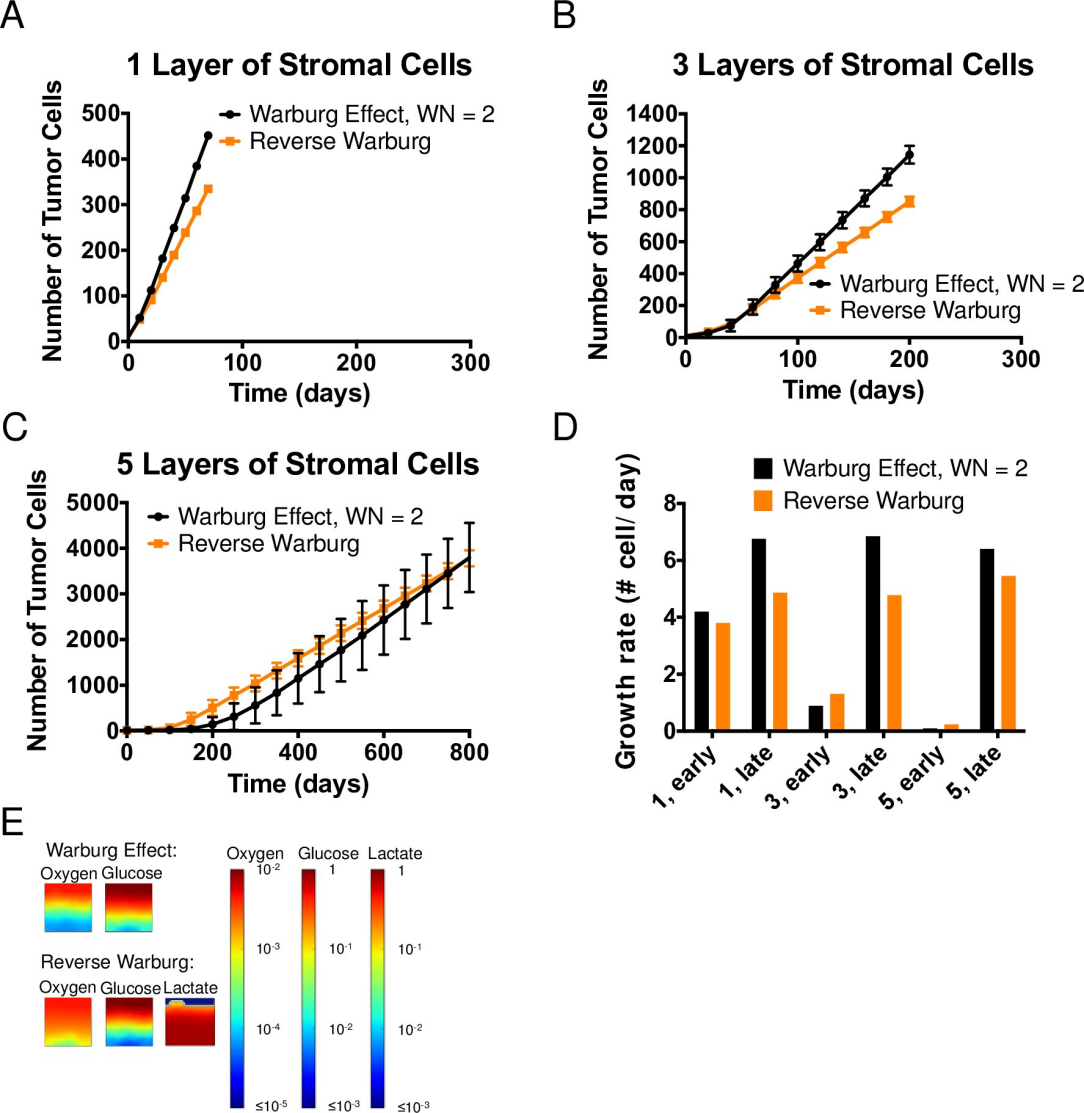
The Reverse Warburg Effect. (A-C) Comparison of growth curves from axial simulations of tumor cells between the Warburg effect (WN = 2) and Reverse Warburg effect when 1, 3 and 5 layers of hijacked stromal cells are seeded between the source and tumor cells. Each time point represents the average of 11 simulations; error bars represent standard deviation. Note differences in vertical scales on plots. (D) Comparison of growth rate of tumor cells at early and late times, extracted from the average growth curves in (A-C). (E) Concentration fields of metabolites in the case of 5 layers of stromal cells at t = 0.

Fig 6A–6C present the comparison of the growth curves of reverse Warburg tumor cells with their hijacked stromal cells (orange) to that of Warburg tumor cells with healthy stromal cells (WN = 2; black; same as black curves in Fig 5A–5C) with 1, 3, and 5 layers of stromal cells. Again, we observe two phases of growth, as in Figs 4B, 5A and 5C. As we concluded previously, the two stages of growth correspond to the pre- and post-breakthrough of tumor cells (see S1–S3 Movies). For 1 layer of stromal cells (Fig 6A), it appears that Warburg tumor cells grew faster in both regimes. On the other hand, for 3 and 5 layers of stromal cells (Fig 6B and 6C), reverse Warburg tumor cells grew faster at early times when they were buried beneath the layers of hijacked stromal cells (i.e., pre-breakthrough), whereas Warburg tumor cells grew faster in the second regime, once breakthrough had occurred.

To investigate these distinctions further, we present the growth rates of tumor cells under these two metabolic scenarios at early and late times in Fig 6D. At early times, we confirm that Warburg tumor cells grew faster than reverse Warburg tumor cells in the case of 1 layer of stromal cells. In this case, the availability of metabolites in tumor-cell compartment was not significantly compromised by diffusion limitations and the consumption by the stromal cells; both phenotypes adopted the aerobic growth regime. According to Eqs 7 and 9, both phenotypes of tumor cells depend on both oxygen and glucose to grow under aerobic conditions. Looking at the yield coefficients on oxygen and glucose shown in Table 1, we can see that reverse Warburg tumor cells have slightly lower yields on oxygen (Y_O2/aer_ = 0.247 g-DW/g for the reverse Warburg phenotype vs. Y_O2/aer_ = 0.349 g-DW/g for the Warburg phenotype, WN = 2) and much higher yields on glucose (Y_Glu/aer_ = 2.68 g-DW/g for the reverse Warburg phenotype vs. Y_Glu/aer_ = 0.098 for the Warburg phenotype, WN = 2). This lower yield on oxygen is a cost of using lactate instead of glucose in the TCA cycle (which in turn affects the ATP production in OXPHOS), underlining the fact that lactate is not an equivalent carbon source to glucose for tumor cells. Therefore, within a favorable metabolic environment (e.g., 1 layer of stromal cells), Warburg tumor cells grew faster due to their better yields on oxygen. Further, upon breaking through the layer of stroma, they obtained direct access to higher concentrations of metabolites, and their growth advantage was amplified, as is reflected in the larger disparity in growth rates at all late times (Fig 6D).

As number of layers of stromal cells increases, the initial tumor growth is more strongly affected by the consumption of metabolites by the stromal cells. This impact is reflected in the opposite trend present in growth rates at early times in the case of 3 and 5 layers of stromal cells (Fig 6D, “3, early”, “5, early”): reverse Warburg tumor cells grew faster than Warburg tumor cells in these cases. We attribute this early-time growth advantage in reverse Warburg tumor cells to the reduced consumption of oxygen by hijacked stromal cells (due to their higher yields on oxygen than healthy stromal cells, Table 1); this effect represents a host (hijacked stromal cells)–parasite (reverse Warburg tumor cells) interaction between the two sub-populations.

We present in Fig 6E the concentration fields of metabolites at t = 0 in the case of 5 layers of stromal cells. At the initial stage of tumor growth, we note that oxygen penetrated deeper into the tissue in the reverse Warburg scenario. Although a large number of stromal cells were present, their adoption of aerobically glycolytic phenotype allowed them to use glucose to generate ATP while producing lactate and allowing oxygen to diffuse into the tumor compartment; this penetration of oxygen allowed the reverse Warburg tumor cells to grow aerobically and thus faster such that they reached breakthrough more quickly (Fig 6C). After breakthrough, due to the lower yields of biomass on oxygen for reverse Warburg tumor cells, the Warburg tumor cells grew faster and eventually outgrew the reverse Warburg tumor cells (Fig 6C–6D).

These observations suggest that the host-parasite relationship between hijacked stromal cells and tumor cells that characterized the reverse Warburg effect can provide growth advantage to tumors that initiate farther away from blood vessels [12], but that this advantage may not persist after the tumor has escaped from its initial, resource-limited environment.

#### Glutamine addiction

**Fig 7:**
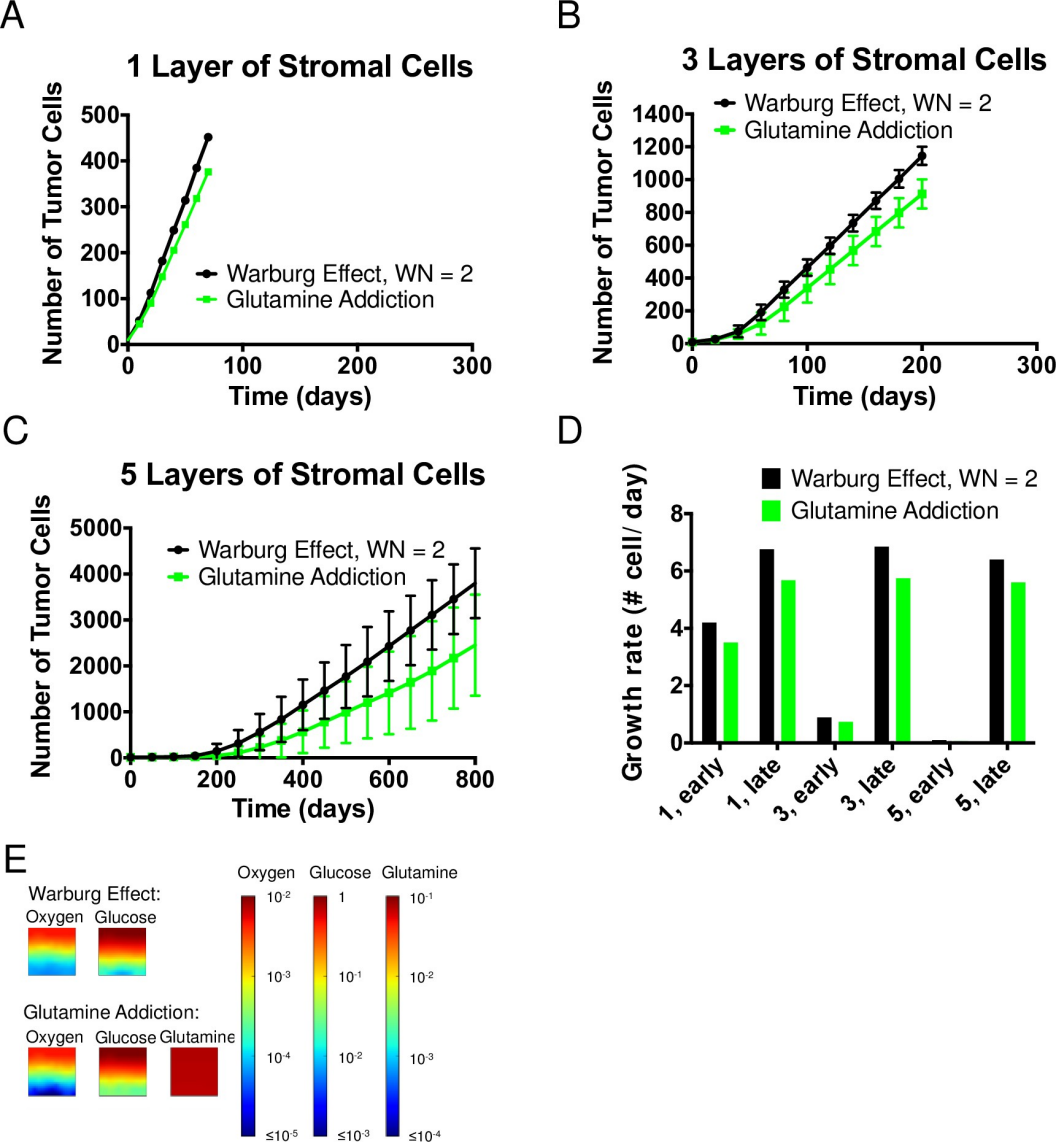
Glutamine addiction. (A—C) Comparison of growth curves from axial simulations of tumor cells between the Warburg effect and Glutamine addiction when 1, 3 and 5 layers of stromal cells are imposed in between the source and tumor cells, respectively. Note differences in vertical scales on plots. Each time point represents the average of 11 simulations; error bars represent standard deviation. (D) Comparison of growth rate of tumor cells at early and late times (see Methods). (E) Concentration fields of metabolites in the case of 5 layers of stromal cells at t = 0.

Fig 7A–7C present the comparison of tumor growth between Warburg tumor (with WN = 2) cells and glutamine-addicted tumor cells. The growth curves of Warburg tumor cells rise above the ones of glutamine-addicted tumor cells in all three cases, indicating a growth advantage in the Warburg scenario. This growth advantage of the Warburg tumor cells increased as tumor cells initiated more deeply in the tissue (from 1 layer (Fig 7A) to 5 layers (Fig 7C) of stromal cells). This observation can be explained as follow: from FBA results shown in Table 1, the use of glutamine by glutamine-addicted tumor cells allows them to uptake much less glucose (higher yields on glucose at 0.148) compared to Warburg tumor cells (lower yields on glucose at 0.098) under aerobic growth regime. However, the required uptake rate of oxygen for glutamine-addicted tumor cells is higher (lower yield coefficient on oxygen at 0.305 vs. 0.349 for Warburg tumor cells in Table 1). This lower yield on oxygen occurs because glutamine passes as α-ketoglutarate via glutamate into the TCA cycle to produce biomass precursors; this pathway leads to lower demand for glucose in the TCA cycle. Subsequently, due to the constraint of WN = 2, there is less lactate production and hence less regeneration of NAD+ from this pathway. Therefore, more oxygen is required to maintain the redox balance in glutamine-addicted tumor cells. As seen in Fig 7D, the higher demand of oxygen by glutamine-addicted tumor cells led to lower growth rates at both the early and late times and for all initial conditions. Additionally, as illustrated in Fig 7E, even in the case of 5 layers of stromal cells, glutamine was never limiting. We attribute this abundance of glutamine to the growth of stromal cells being independent of glutamine and the initiation site of glutamine-addicted tumor cells being oxygen depleted. We conclude that oxygen and glucose remain the limiting metabolites in this metabolic scenario.

Based on our assumptions and results, we infer that for a typical value of WN = 2, the role of glutamine in anaplerosis does not confer improved fitness to tumor population relative to Warburg effect in a geometrically confined microenvironment. Therefore, unless glutamine-addicted tumor cells utilize oxygen at a similar efficiency as Warburg tumor cells, for example by adopting a metabolic phenotype with higher Warburg Number than Warburg tumor cells (e.g., being MYC-positive may allow tumor cells to have even higher glycolytic rate [60]), the experimentally observed glutamine addiction in diverse tumor cell types cannot be explained by its role in supplying carbon to the TCA cycle. Therefore, we suggest that some other biological roles of glutamine, not included in the current model, must underlie this phenomenon.

## Discussion

### Warburg effect improves tumor fitness in resource-limited microenvironment

Within our scope of study of the Warburg effect through the multi-scale modeling approach (Figs 4 and 5), we confirmed a common hypothesis that Warburg effect impacts tumor cell fitness in metabolically limited microenvironments [64]. Interestingly, our predictions suggest that there may exist an optimal level of Warburg effect (reflected by the ratio of pyruvate fluxes to lactate and to the TCA cycle; the Warburg Number) for tumor cells to adopt depending on the details of the metabolic microenvironment in which the tumor cells initiate. This observation may help explain the experimentally observed phenotypic heterogeneity in cancer metabolism [65,66]. Such adaptation could occur via modification of the fluxes of pyruvate, for example with changes in enzymatic rates along either the TCA cycle or glycolytic pathways. From an ecological perspective, our predictions indicate that Warburg effect may provide a basis for adaptation of tumor cells to different environmental metabolic stresses [67].

### Reverse Warburg effect provides tumor growth advantage depending on the initial microenvironment

For the reverse Warburg effect scenario (Fig 6), we provide the first mathematical description of the multi-cellular metabolic interactions proposed by Sotgia *et al*. [55]. We used our framework to explore the intracellular and multicellular consequences of reverse Warburg effect due to the interaction between glycolytic stromal cells (hijacked stromal cells) and lactate-consuming tumor cells (reverse Warburg tumor cells). We predict that the hijacked stromal cells have higher yields on oxygen than healthy stromal cells (Fig 2B i) and Table 1). This information confirmed the intuitive proposal of Sotgia *et al*. that the hijacked stromal cells assist in the growth of tumors that initiate deep within the stroma by allowing more oxygen to penetrate into the tumor compartment (Fig 6C–6E). We further note that, due to the adaptive character of reverse Warburg tumor cells, they are not sensitive to local lactate concentration in aerobic growth regimes (term 1 and 2 on the right-hand side of Eq 9 can be combined); this characteristic means their aerobic growth remains limited by oxygen and glucose only. Additionally, due to the utilization of lactate as carbon source in energy production in these tumor cells, their yields on oxygen is lower compared to tumor cells in the scenario of Warburg effect (requiring more oxygen for the same mole of carbon consumed). Therefore, the reverse Warburg effect leads to slower growth in favorable metabolic microenvironment (i.e., abundant source of metabolites available). However, when tumors initiate in microenvironments where resources are significantly reduced, the host-parasite relationship implied by the reverse Warburg effect (via cooperative utilization of oxygen between hijacked stromal cells and tumor cells) can provide growth advantage to tumors. Given that such growth advantage depends on the detailed structure of the metabolic microenvironment, we suggest that one must use a multi-scale framework like the one presented here to investigate the implications of these metabolic scenarios.

### Glutamine addiction as a hallmark of cancer metabolism

In the exploration of glutamine addiction (Fig 7), we defined the metabolic phenotype by hypothesizing that glutamine addiction coexists with Warburg effect. This hypothesis led us to propose a coupled contribution to biomass synthesis of tumor cells from glucose and glutamine as joint carbon sources. Specifically, we aimed to explore the role of glutamine in anaplerosis (as a carbon source to replenish the TCA cycle). We demonstrated with FBA that under our interpretation, glutamine addiction led to an increase uptake of oxygen (i.e., lower yield on oxygen) in glutamine-addicted tumor cells to maintain their redox balance and to meet the energy demand; this lower yield on oxygen represents a cost of using glutamine in the TCA cycle. We see the impact of this lower yield on oxygen in the reduced growth rate of glutamine-addicted tumor cells relative to Warburg tumor cells. We thereby conclude that glutamine addiction via the process of anaplerosis does not confer an advantage to the overall tumor growth primarily due to the strong dependence on oxygen. We argue that glutamine is not an effective alternative carbon source because tumor cells remain limited by glucose and oxygen.

Our study constrains future considerations of the roles of glutamine addiction in tumor growth by clearly demonstrating that the anaplerotic pathway cannot, alone, provide a growth advantage to tumors. With our focus on the anaplerotic role of glutamine using a simplified metabolic network, we did not account for other roles of glutamine in cellular demand explicitly [68,69]. For example, glutamine is known to be an important nitrogen source in nucleic acids and amino acids synthesis [57,70,71]. Additionally, glutamine contributes to the pool of metabolites that maintains NADPH/NADP+ balance [69,72] and to produce glutathione as an antioxidant to help the cell resist oxidative stress during rapid metabolism [70,72]. We conclude that a more detailed investigation that accounts for the multi-scale implications of these additional pathways is needed in the future.

### Multi-scale modeling framework

With our approach, the growth curves captured in our spatially resolved model (a slow growth regime followed by a fast unidirectional linear growth) are compatible with the experimentally observed growth of avascular solid tumors [63,73]. Previous studies attributed the linear growth regime observed at late-time tumor growth to available space for growth and cell diffusion at the edge of the tumors [73,74]. Here, our simulations and analysis indicate that this effect can be entirely explained by diffusion limitations of metabolites.

In our exploration of Warburg and reverse Warburg effect, our approach provided a basis for exploring the heterogeneity in metabolic phenotypes that has been suggested by recent experiments [65,66]. For example, the crossover of growth rates that we observed from early to late times (Figs 5C and 6C) suggests that adaptation of metabolic phenotypes (e.g., from high to intermediate WN or from RW to Warburg) could improve overall growth potential of tumors.

In parallel with experimental approach, computational tools allow for high throughput investigation of hypotheses that are emerging rapidly in the field of cancer study [24,29,33,34,68,75,76]. Particularly, a multi-scale modeling framework such as the one presented here can provide a basis for predicting cell-level to tissue-scale response to therapeutic interventions. For example, the action of inhibitors of key regulators of cellular metabolism such as PI3K [77] can be accounted for in FBA as flux constraints (e.g., a reduced upper bound on glycolytic flux); the obtained uptake rates of metabolites could then be propagated through to the tissue-scale ABM in our framework in order to examine the effect on tumor growth at the population scale.

We finish by emphasizing that our interpretations of the three metabolic scenarios studied here are not unique either with respect to the choices of constraints and objectives imposed for FBA or the details of the cellular configurations within our simulations. Our modeling framework can accommodate a large diversity of hypotheses and should serve as a powerful tool with which to evaluate emerging ideas and experimental observations from the rapidly evolving field of cancer metabolism.

## Methods

### Intracellular–Modeling biomass production using a stoichiometric model

To capture the intracellular details of different metabolic phenotypes of cells, we adopt the well-established framework of FBA.

In our study, the central carbon metabolism of human was constructed with 140 reactions and 92 metabolites (S1 Fig). Of those 140 reactions, 34 consist of boundary exchange of metabolites such as uptake and secretion, 26 consist of mitochondrial exchange of metabolites with the cytosol, 1 is the biomass template reaction, 1 reaction for maintenance, and the 78 remaining reactions are transformations of metabolites that occur in the cytosol and mitochondrion. The biomass template reaction for growth in the human model was adapted from the Shlomi *et al*.’s genome-scale model [20]. Shlomi *et al*.’s biomass template reaction consists of 30 biomass compounds including amino acids (0.78 g/g-DW), nucleotides (0.06 g/g-DW), and lipids (0.16 g/g-DW). These biomass requirements were combined and reduced to their upstream precursors for simplification in our biomass template reaction. For example, stoichiometric equivalence of ribose-5-phosphate, the precursor of nucleic acids, was used in place of nucleotides in their final form. For the maintenance rate, we first sampled a range of values from 0 to 10 mmol ATP/g-DW-hr [78–80] and concluded that the overall qualitative trend of our FBA results was not affected by this choice. Therefore, for simplicity, a maintenance rate of 5 mmol/g-DW-hr is used consistently for all cell types. This maintenance rate represents 73% of the total energy expenditure, comparable to what was previously reported for mammalian cells, which is 65% [80]. Using our reduced biomass function, our glucose yields (Y_G/n_) matched closely with that of Shlomi and coworkers [20]. For example, within the metabolic phenotype of WN = 0, at the same growth rate range and maintenance rate of 0, the yield coefficient (specific growth rate per glucose) of our reduced order model (0.0984 g-DW/mmol) was within 4% of that found with Shlomi *et al*.'s genome scale model (0.094 g-DW/mmol).

### Metabolic phenotypes in hypoxic and hypoglycemic conditions

Under hypoxic conditions (C_O_ << K_O_), we assumed a quiescent phenotype for all cell types. To capture the hypoxic condition, we minimized the oxygen uptake rate while maintaining a growth rate of 1 ×10^−6^ hr^-1^ to represent the quiescent state.

For tumor cells in the metabolic scenarios of reverse Warburg effect and glutamine addiction, we used a quiescent phenotype for tumor cells under hypoglycemic conditions (C_G_ <<K_G_). We achieve this condition in FBA by minimizing glucose uptake while allowing uptake of lactate or glutamine respectively and constraining growth rate to be 1×10^−6^ hr^-1^.

### Use of agent-based simulation tool at multicellular scale—iDynoMiCS

iDynoMiCS is an individual-based modeling platform originally built for the study of microbial biofilms [39]. It allows computation of diffusion-reaction kinetics at individual cell level and has multiple built-in kinetic mechanisms, including Monod forms as in Eqs 5–10. Additionally, iDynoMiCS treats the cell movement through two mechanisms: displacements due to pressure-induced convection at the global scale based on Darcy’s law, and sterically induced displacements that avoid overlapping during the expansion and division of neighboring cells at a local scale. During a simulation, the pressure that is directly proportional to the rate of biomass generation or degradation is computed first to induce global convection, followed by the computation of “shoving” (random displacement) at local scale; these displacements are selected by a relaxation algorithm to avoid steric overlap. The shoving mechanism is propagated through all cells until the number of cells that are still moving is negligible, and leads to local random displacements of cells [39].

In our case, since we are explicitly interested in studying how diffusion-reaction kinetics impact the tumor growth under various hypotheses on cancer metabolism with no specific consideration of molecular guidance for cell movements, the random, local cell motion provided by iDynoMiCS serves as a reasonable approximation of cell dynamics within the tissue [81]. The 2-D simulation domain is discretized into a square grid on which the reaction-diffusion equation is solved by finite difference at each time step (Eqs 2 and 4). The domain is also divided into two compartments: the “tank” and the “biofilm”. The tank serves as the source of metabolites; we interpret this compartment to be the blood stream with which the tissue exchanges nutrients. The “biofilm” defines the tissue where the metabolites undergo diffusion and reaction; the local reaction rate for each metabolite is set by the density and metabolic character of the cells in the grid element. A boundary layer defines the resistant to diffusive mass transfer between the blood stream (“tank”) and the cells (“biofilm”). In our axial simulations, we allowed the exchange of metabolites only at the top of the domain by having zero-flux boundary condition at the bottom of the domain and periodic boundary conditions on the sides and in the 3^rd^ dimension (S3 Fig). We set the concentrations of metabolites in the “tank” at their physiological concentrations in human blood stream (S1 Table). We selected a grid size for solving reaction-diffusion process to match individual mammalian cell size (~10 μm, [82]) and a boundary layer thickness, h, to represent the thickness of the vascular endothelium (S1 Table). The size of the cell was used to determine the density of the cell based on dry cell mass (S1 Table). With the density of the cell fixed, we calculated the spherical volume of the cell from biomass growth by conservation of mass. This volume was then used to calculate the diameter of cells at each time step. The calculated diameter at each time step was then used to compare to a threshold value to determine the division of the cell. Once the computational domain was defined, we then specified the reactions that govern the cell growth. In each reaction, we chose parameters such as half saturation constant (S1 Table). Together with parameters such as diffusion coefficients and physiological concentrations of metabolites obtained from the literature, we checked that the calculated value of the Krogh length (e.g., ~40μm for oxygen) was in the right range for mammalian tissue.

### Calculation of Krogh length

In the calculation of Krogh length, we treat the tissue as a continuum and represent consumption of oxygen and glucose as being zero^th^ order within the steady state reaction-diffusion equation. We calculated the Krogh lengths to determine the limiting metabolite in tumor cell growth in different metabolic scenarios (i.e., different WNs, Fig 5F). The Krogh lengths represent the typical depth of penetration of metabolites into the tumor compartment. We omitted the consumption contributed by anaerobic growth of the cells by assuming the metabolites get completely depleted before the cells switch to anaerobic growth regime. The calculation of Krogh lengths is illustrated in S2 Fig. The metabolite with shorter Krogh length will play a more significant role in determining the growth dynamics of tumor cells.

### Extraction of early- and late-time tumor growth rates

In Figs 5–7, we evaluated early-time growth rates as the initial slope of the growth curves by taking the difference of the averaged number of tumor cells for the first two outputs of simulation and dividing by the time interval. The time intervals are 10 days, 20 days and 50 days for the cases of 1, 3 and 5 layers of stromal cells for all three metabolic scenarios.

Late-time growth rates were obtained in a similar fashion but evaluated at different time intervals due to the difference in breakthrough times in different cases. A growth over 30 days between the time points 30 and 60 days was used in the case of 1 layer of stromal cells. A growth over 80 days between the time points of 120 and 200 days was used for calculation of late-time growth rates in the case of 3 layers of stromal cells. A growth over 200 days between the time points of 400 and 600 days was applied to the calculation of late-time growth rates in the case of 5 layers of stromal cells. These choices of time ranges were applied consistently in all three metabolic scenarios.

## Supporting information

S1 Fig Full mammalian central metabolic network used in flux balance analysis. (PDF)

S2 Fig Calculation of krogh lengths based on diffusion and zero^th^ order consumption by tumor cells. (PDF)

S3 Fig Boundary conditions in axial simulations. (PDF)

S1 Table Parameters used in iDynoMiCS for Monod kinetics of cellular growth and reaction-diffusion of metabolites. (DOCX)

S1 Movie Radial tumor growth with 1 layer of healthy stromal cells. (MP4)

S2 Movie Radial tumor growth with 3 layers of healthy stromal cells. (MP4)

S3 Movie Radial tumor growth with 5 layers of healthy stromal cells. (MP4)

S1 Octave files for flux balance analysis (ZIP)
