# Supplementary figures and images for: Multi-scale computational study of the Warburg effect, reverse Warburg effect and glutamine addiction in solid tumors
Source: PLoS Comput Biol. 2018 Dec 7;14(12):e1006584. doi: 10.1371/journal.pcbi.1006584 (PMC6285468; doi:10.1371/journal.pcbi.1006584)

Extracellular Metabolites

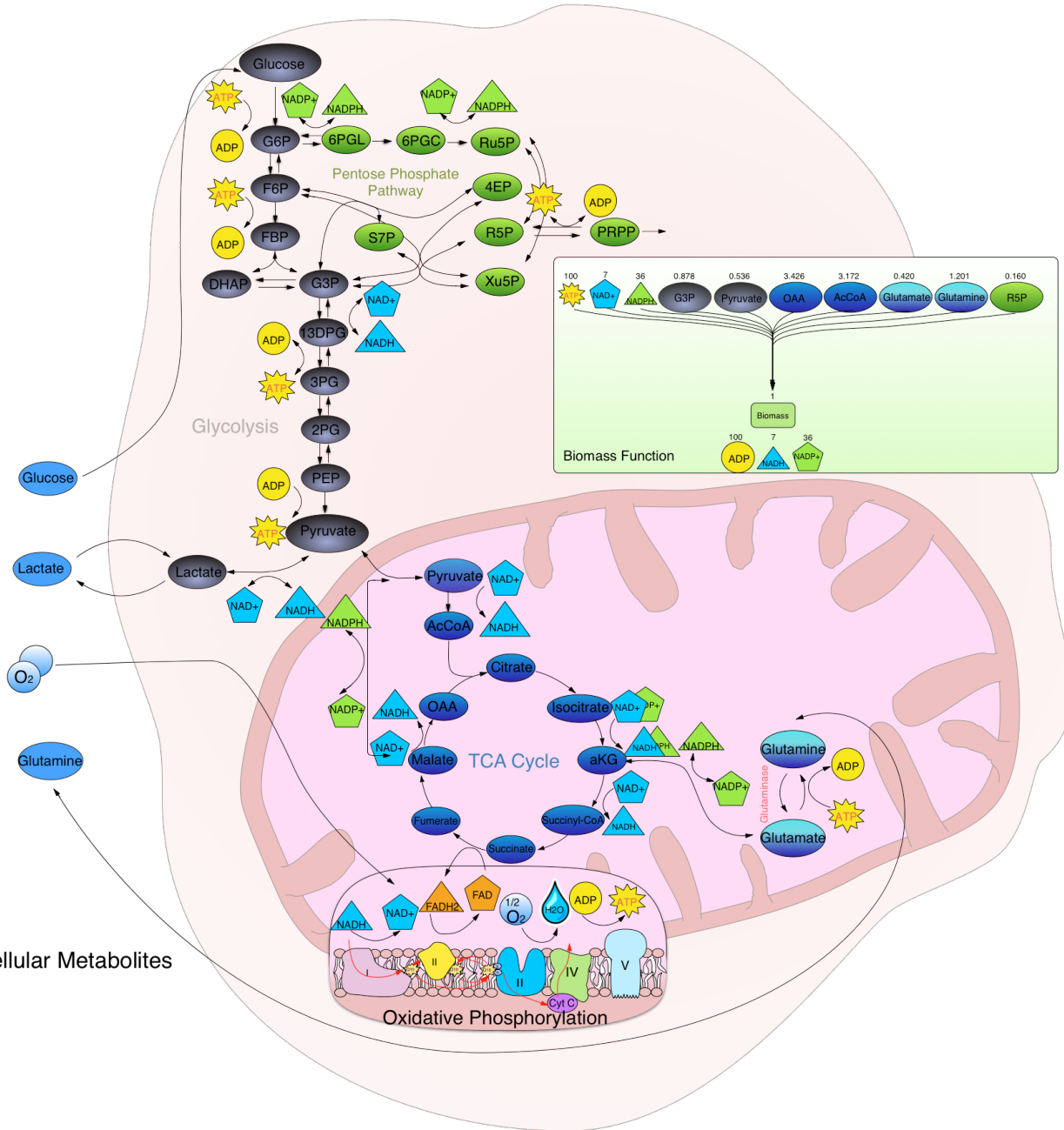

Supplement: S1 Fig — (PDF) [file pcbi.1006584.s001.pdf]

①  $x = 0, C = \text{Source}$

②  $x = \lambda_K, C_i = 0$

③  $x = \lambda_K, \frac{dC_i}{dx} = 0$

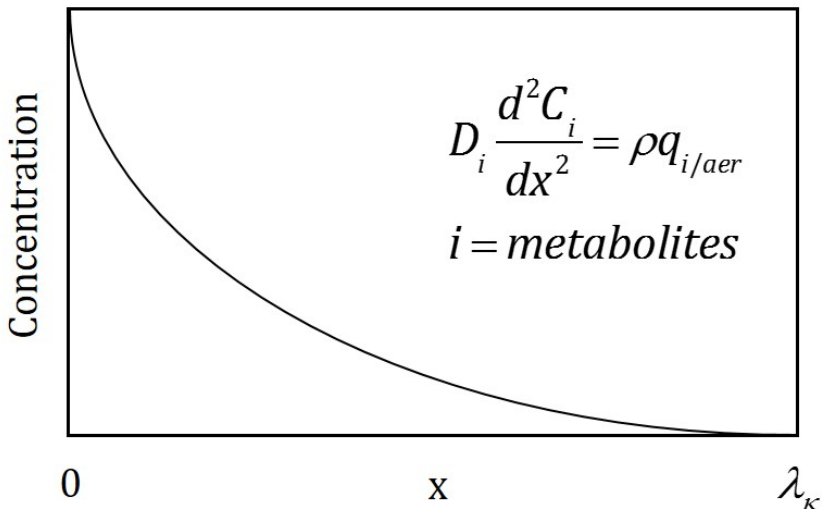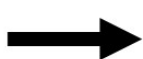

$\lambda_K$

Supplement: S2 Fig — (PDF) [file pcbi.1006584.s002.pdf]

bulk

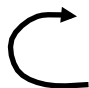

periodic

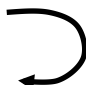

periodic

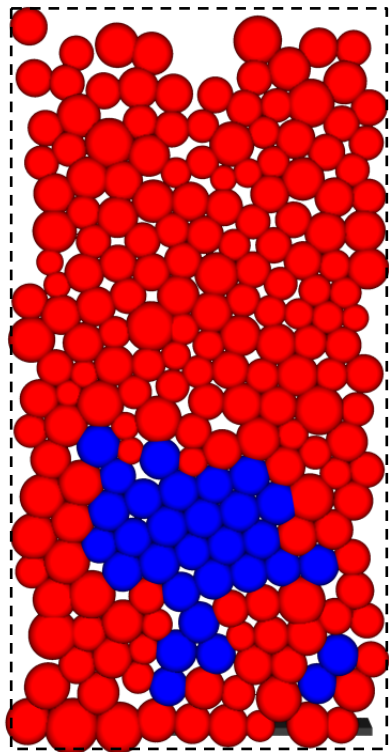

zero flux

Supplement: S3 Fig — (PDF) [file pcbi.1006584.s003.pdf]
