# Supplementary material for: Multi-scale computational study of the Warburg effect, reverse Warburg effect and glutamine addiction in solid tumors
Source: PLoS Comput Biol. 2018 Dec 7;14(12):e1006584. doi: 10.1371/journal.pcbi.1006584 (PMC6285468; doi:10.1371/journal.pcbi.1006584)
Supplement: S1 Table — (DOCX) [file pcbi.1006584.s004.docx]

| Parameter | | Description | Value | Units | Reference |
| --- | --- | --- | --- | --- | --- |
| D_O2_ | Diffusion coefficient of oxygen | | 3.11×10^-4^ | m^2^/day | [1] |
| D_Glu_ | Diffusion coefficient of glucose | | 2.16×10^-5^ | m^2^/day | [2] |
| D_Lac_ | Diffusion coefficient of lactate | | 2.5×10^-5^ | m^2^/day | [3] |
| D_Gln_ | Diffusion coefficient of glutamine | | 2.32×10^-5^ | m^2^/day | Estimated based on Stokes-Einstein using MW of glucose and glutamine |
| K_O_ | Half saturation constant of oxygen | | 4.6×10^-4^ | g/L | see text |
| K_G_ | Half saturation constant of glucose | | 0.1 | g/L | see text |
| K_L_ | Half saturation constant of lactate | | 0.1 | g/L | see text |
| K_N_ | Half saturation constant of glutamine | | 7.3×10^-3^ | g/L | see text |
| S_O_ | Physiological concentration of oxygen | | 4.6×10^-3^ | g/L | based on Henry’s law at 37 °C and 100 mmHg |
| S_G_ | Physiological concentration of glucose | | 1 | g/L |  |
| S_L_ | Physiological concentration of glucose | | 0 | g/L |  |
| S_N_ | Physiological concentration of glucose | | 7.3×10^-2^ | g/L | [4] |
| ρ | dry mass density of the cell | | 2000 | g/L | based on 3.5 ng/cell of which 30% is dry mass and radius of 10 μm |
| h | Boundary layer thickness in iDynomics | | 15 | μm | see text |

References:

1. MacDougall JD, McCabe M. Diffusion coefficient of oxygen through tissues. Nature. 1967;215: 1173–1174. doi:10.1038/2151173a0

2. Tuchin V, Genina E, Larin K. Measurement of Glucose Diffusion Coefficients in Human Tissues. 2008. pp. 587–621. doi:10.1201/9781584889755.ch19

3. Bassi AS, Rohani S, Macdonaid DG. F ). 1987;30: 794–797. Available: http://onlinelibrary.wiley.com/store/10.1002/bit.260300614/asset/260300614_ftp.pdf?v=1&t=j8nxzvhm&s=ba077ffa86254e77ccb04c8009f9017ee67336fa

4. Shimmura C, Suda S, Tsuchiya KJ, Hashimoto K, Ohno K, Matsuzaki H, et al. Alteration of Plasma Glutamate and Glutamine Levels in Children with High-Functioning Autism. Deli MA, editor. PLoS One. Public Library of Science; 2011;6: e25340. doi:10.1371/journal.pone.0025340
